# Simulating and Predicting Adsorption of Organic Pollutants onto Black Phosphorus Nanomaterials

**DOI:** 10.3390/nano12040590

**Published:** 2022-02-09

**Authors:** Lihao Su, Ya Wang, Zhongyu Wang, Siyu Zhang, Zijun Xiao, Deming Xia, Jingwen Chen

**Affiliations:** 1 Key Laboratory of Industrial Ecology and Environmental Engineering (Ministry of Education), Dalian Key Laboratory on Chemicals Risk Control and Pollution Prevention Technology, School of Environmental Science and Technology, Dalian University of Technology, Dalian 116024, China; su_lihao@mail.dlut.edu.cn (L.S.); yaanne@mail.tsinghua.edu.cn (Y.W.); wzy1989@mail.dlut.edu.cn (Z.W.); xzj960729@mail.dlut.edu.cn (Z.X.); xiadm@mail.dlut.edu.cn (D.X.); 2 Key Laboratory of Pollution Ecology and Environmental Engineering, Institute of Applied Ecology, Chinese Academy of Sciences, Shenyang 110016, China

**Keywords:** black phosphorus, nanomaterial, pp-LFER, density functional theory, molecular dynamic simulation

## Abstract

Layered black phosphorus (BP) has exhibited exciting application prospects in diverse fields. Adsorption of organics onto BP may influence environmental behavior and toxicities of both organic pollutants and BP nanomaterials. However, contributions of various intermolecular interactions to the adsorption remain unclear, and values of adsorption parameters such as adsorption energies (*E*_ad_) and adsorption equilibrium constants (*K*) are lacking. Herein, molecular dynamic (MD) and density functional theory (DFT) was adopted to calculate *E*_ad_ and *K* values. The calculated *E*_ad_ and *K* values for organics adsorbed onto graphene were compared with experimental ones, so as to confirm the reliability of the calculation methods. Polyparameter linear free energy relationship (pp-LFER) models on *E*_ad_ and log*K* were developed to estimate contributions of different intermolecular interactions to the adsorption. The adsorption in the gaseous phase was found to be more favorable than in the aqueous phase, as the adsorbates need to overcome cohesive energies of water molecules onto BP. The affinity of the aromatics to BP was comparable to that of graphene. The pp-LFER models performed well for predicting the *E*_ad_ and *K* values, with external explained variance ranging from 0.90 to 0.97, and can serve as effective tools to rank adsorption capacities of organics onto BP.

## 1. Introduction

Layered black phosphorus (BP) has become a nanomaterial star since 2014 in diverse fields, including optoelectronics, energy storage, sensor and biomedical applications, etc. [1,2,3]. Because of its superior semiconductor properties [4], BP has been igniting an upsurge in laboratory investigations. Recently, with developments in production techniques, the production costs of BP have been largely reduced to <1 $g^−1^ [5]. In the foreseeable future, BP or derivative products are promising for massive industrial productions, and commercial or engineering applications.

BP possesses a large theoretical specific surface area of 2400 m^2^ g^−1^ close to that of graphene [6], and exhibits an extremely high drug-loading ability [7]. Accordingly, BP, once released into the environment, may adsorb a variety of organic and inorganic pollutants. The interactions between BP and organic/inorganic pollutants may have impacts on environmental behavior, bioavailability, toxicology and ecological risks of both BP and organic/inorganic pollutants [8,9,10], as can be inferred from previous studies concerning adsorption of organic chemicals onto carbon nanomaterials such as carbon nanotubes and graphene [11,12,13]. It is known that BP can be oxidized by oxygen that attacks the lone-pair electrons of phosphorus [14]. The adsorption of organic chemicals may protect BP against oxidation by interacting with the lone-pair electrons and influence the environmental lifetime of BP. Therefore, it is important to investigate the adsorption of organic chemicals onto BP for understanding and assessing their environmental risks, and it is also of interest to understand the distinctions in adsorption mechanisms (i.e., intermolecular interactions) between BP and the widely investigated graphene [15,16].

Adsorption energies (*E*_ad_) and equilibrium constants (*K*) are essential parameters characterizing the adsorption of chemicals onto nanomaterials. Due to the large and ever-increasing number of environmental organic chemicals, it is unrealistic and impossible to exhaustively determine the values of these parameters experimentally. Quantitative structure-activity relationship (QSAR) models may serve as alternatives to the empirical values of the parameters [16,17,18,19,20]. To the best of the authors’ knowledge, there have been no available QSAR models that can be employed to predict the adsorption parameters of organics onto BP.

Compared with the comprehensive experimental adsorption data onto carbon nanomaterials (e.g., carbon nanotubes, graphene and graphene oxides) [15,21,22,23,24,25,26,27], adsorption of only nine organic compounds (benzene, dioxane, cyclohexane, acetonitrile, nitromethane, acetone, tetrachloromethane, methylene blue and congo red) onto BP has been experimentally investigated to obtain *E*_ad_ or *K* so far [6,28]. Thus, the development of QSARs for predicting adsorption onto BP is seriously impeded by a realistic problem of insufficient experimental data on *E*_ad_ or *K*. This can be solved by computations based on density functional theory (DFT) or molecular dynamics (MD) [24,25,29,30].

DFT can accurately calculate *E*_ad_ of a static adsorption configuration and has been successfully applied to probe adsorption of different organic chemicals onto carbon nanomaterials [24,25,31]. Lazar et al. [24] found that the DFT calculated *E*_ad_ values for adsorption of seven organic molecules onto graphene were in excellent agreement with the experimental ones. MD is capable of providing dynamic evolution of the interactions between nanomaterials and organic chemicals in aqueous environments, which is helpful for explaining the adsorption mechanisms. With MD calculation, Tang et al. [32] found aromatic compounds exhibited a strong preference for the edges and wrinkles of graphene oxides. Besides, MD integrated with an adaptive biasing force (ABF) method has been proved to be reliable and efficient in calculating *K* values and has been successfully used to evaluate adsorption affinities of different organic compounds onto carbon nanomaterials [29,30].

Theoretically, adsorption of organic compounds onto BP is governed concurrently by multiple intermolecular interactions, similar to the adsorption of various organics onto carbon nanomaterials, such as electrostatic, van der Waals, hydrophobic interactions and hydrogen bonds [12,15,16]. Polyparameter linear free energy relationships (pp-LFERs), which form a theoretical basis of QSARs and employ a set of Abraham molecular descriptors (*E*, *S*, *A*, *B*, *V* and *L*) [33], have been successfully employed to elucidate relative contributions of the different interactions to the overall adsorption onto carbon nanomaterials [16,18,34]. However, contributions of the different interactions to the overall adsorption onto BP have not been estimated yet.

In this study, the DFT and MD methods were evaluated by comparing the calculated *E*_ad_ and *K* values with empirical ones for the adsorption of organics onto graphene. *E*_ad_ and *K* values of a series of organic compounds (aliphatic hydrocarbons, benzene and its derivatives, polycyclic aromatic hydrocarbons) adsorbed onto BP were calculated by the DFT and MD methods, respectively. The objectives of this study were three-fold: (1) to develop pp-LFER models on *E*_ad_ and *K* from the DFT or MD results so as to fill the data gap of BP adsorption, and (2) to elucidate adsorption mechanisms of environmental organic chemicals onto BP by quantifying the relative contributions of the various interactions to the overall adsorption and (3) to unveil the differences in adsorption between BP and graphene for organic compounds by a comprehensive comparison of the pp-LFER models.

## 2. Computational Methods

### 2.1. DFT Calculation

As listed in Table S1 in supporting information (SI), 41 aliphatic and aromatic compounds with diverse functional groups were selected as model adsorbates. As these compounds were often selected in previous adsorption studies on carbon nanomaterials [16,30], the comparison on the different adsorbing nanomaterials can be implemented.

A 6 × 5 × 1 BP supercell containing 120 phosphorus atoms was built as an adsorbent model. Periodic boundary conditions were applied in the adsorption system. A box of 19.88 × 21.87 × 22.16 Å^3^ was adopted to eliminate interactions of the adsorbates with BP in neighboring periodic structures. Similar phosphorene models have been implemented to study their interactions with 2,3,7,8-tetrachlorodibenzo-*p*-dioxin and amino acids [35,36]. The structural details about the graphene model were given in the Supplementary Materials.

All the DFT calculation was carried out with the Dmol^3^ program [37,38] in the Materials Studio package. A Perdew-Burke-Ernzerhof generalized gradient approximation (GGA-PBE) [39] method was employed to calculate exchange-correlation energies, and van der Waals interactions were treated by an empirical correction method proposed by Grimme (DFT-D2) [40]. These methods have also been well implemented to investigate the adsorption of graphene and boron nitride [16,41]. A double-numeric quality with a polarization function (DNP) basis set (comparable to the 6-31G** basis set) [42,43] was used. DFT semi-core pseudopotentials treatments were employed to deal with the relativistic effects of bromine atoms [44]. A 4 × 4 × 1 *k*-grid mesh was adopted for the Brillouin zone integration [45] that was required for calculating the energy by DFT. Conductor-like screening model (COSMO) [46], an implicit solvation model, was utilized to mimic aqueous environments by employing a dielectric constant of 78.54 F∙m^−1^.

*E*_ad_ was calculated as:

*E*_ad_ = *E*_complex_ − *E*_adsorbent_ − *E*_adsorbate_ (1)

where *E* stands for the total energy of a configuration, including kinetic energy, static potential energy, Coulomb energy and exchange-correlation energy [47,48]; *E*_complex_, *E*_adsorbent_, and *E*_adsorbate_ are total energies of the adsorption complexes, adsorbents (i.e., BP and graphene) and adsorbates, respectively. The more negative is the *E*_ad_, the stronger is the interactions between the adsorbents and adsorbates.

### 2.2. MD Simulation

The MD calculations were carried out with NAMD 2.12 [49]. Force field parameters for the adsorbates were generated according to the CHARMM General Force Field (CGenFF) [50] parameters with the ParamChem Web interface [51,52]. The generated parameter files contain “penalty scores” that indicate whether the parameters can be directly used. When the penalty value is >10, the parameters need to be further optimized [52]. The Force Field Toolkit (ffTK) [53] used for developing CHARMM-compatible parameters was employed to further refine the parameters for compounds with penalty scores of >10.

Force field parameters of BP were derived from Blankschtein et al. [54], which accurately reproduced experimental crystal structures and mechanical properties. The force field parameters and structural information for graphene were detailed in the Supplementary Materials. A TIP3P water model [55] adopted in the CHARMM force field was employed for simulating aqueous environments.

All the simulation was run with a 2 fs time-step in the isothermal-isobaric ensemble at 1.01325 × 10^5^ Pa and 300 K by the Langevin piston and Langevin thermostat method [56]. The particle-mesh Ewald algorithm [57] with 1.2 Å grids was employed to deal with electrostatic interactions. A cutoff for truncating non-bonded interactions was set to 9 Å according to previous adsorption simulations of compounds onto graphene and carbon nanotubes [26,30]. The system was subject to energy minimizations of 10^4^ steps and equilibrations of 0.5 ns. An MD integrated with the ABF method [58,59], as implemented in the Colvars module [60] of NAMD 2.12, was employed to compute the potential of mean force (PMF) to obtain the free energies *G*(*Z*) along a transition coordinate (*Z*). As indicated by Figure S1a, *Z* was defined as the vertical distance between the center of mass for the adsorbates and of the first layer of BP [26]. VMD 1.9.3 was adopted for the analysis and visualization [61].

Before the MD simulation, one adsorbate molecule was arranged on the top of a three-layer BP adsorbent in a periodic box of 3.6 × 3.6 × 6.5 nm^3^ (Figure S1b). According to previous adsorption simulations about the carbon nanomaterials [26,30], *Z* was estimated to range from 3 to 15 Å in this study. A single window with the *Z* interval was adopted as the sampling space. The force was sampled in bins of 0.05 Å width along *Z*. *G*(*Z*) was normalized to zero at a distance of 14 ≤ *Z* ≤ 15 Å, where the energies reached a plateau as there were no interactions between BP and the adsorbates (Figure S2). The convergence of *G*(*Z*) in the free energy calculation was checked as detailed in the Supplementary Materials.

The difference in the adsorption free energy (Δ*G*_MD_) that represents the energy required to detach an adsorbate vertically from the surface, was calculated as:

Δ*G*_MD_ = *G*(*Z*_min_) − *G*(*Z*_far_) (2)

where *G*(*Z*_min_) and *G*(*Z*_far_) stands for the minimum *G* value, and the value for the adsorbate far from the nanomaterial surface (Figure S2), respectively.

*K* (mL/g) was calculated according to the following equation that has been applied to compute *K* of organic compounds onto carbon nanomaterials [29,30]:

(3) $$K=S_{A}\int^{\;}e^{-G(Z)/kT}dZ$$

where *k* is Boltzmann constant (*k* = 1.381 × 10^−23^ J K^−1^), *T* is temperature (*T* = 300 K), and *S*_A_ represents specific surface areas of adsorbents. The theoretical specific surface areas (*S*_A(BP)_ = 2400 m^2^ g^−1^; *S*_A(graphene)_ = 2630 m^2^ g^−1^) [6,62] were adopted.

### 2.3. pp-LFER Modeling

The values of the Abraham descriptors were collected from the UFZ-LSER database [63]. The model adsorbates with the calculated log*K* and |*E*_ad_| values were randomly split into a training and a validation set (Table S1) with a ratio of 4:1. Multiple linear regressions (MLR) were utilized for establishing the pp-LFER models. The goodness of fit and robustness of the model were evaluated by adjusted determination coefficient (*R*_adj_^2^), root-mean-square error (*RMSE*), leave-one-out cross-validated *Q*^2^_LOO_ and *k*-fold cross-validation *Q*^2^_kfold_ (5 fold, 5000 repetitions) [64]. External explained variance (*Q*^2^_ext_) [64] was used to estimate the model predictive power. Application domains for the models were characterized with Williams plots [65].

## 3. Results and Discussion

### 3.1. Reliability of Computational Methods

**Figure 1:**
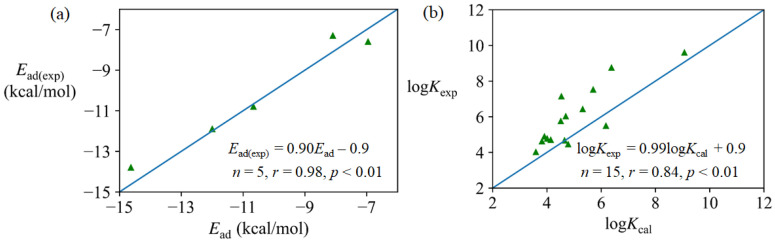
Linear correlations between experimental and calculated values: (**a**) experimental *E*_ad(exp)_ and calculated *E*_ad_ values for adsorption of acetonitrile, benzene, 1,4-dioxane, ethanol and toluene onto graphene in gaseous phase; (**b**) experimental log*K*_exp_ and MD calculated log*K*_cal_ values for adsorption of 15 aromatic compounds onto graphene in the aqueous phase.

As experimental data (*E*_ad_ and *K*) on adsorption of BP are of deficiency, reliabilities of the DFT and MD methods were evaluated by taking the widely investigated graphene as an example. As can be seen from Figure 1a, the DFT calculated *E*_ad_ values linearly correlate (*r* = 0.98) with the experimental *E*_ad(exp)_ values [24,66] for five compounds adsorbed onto graphene (Table S2), and the slope is close to 1. The absolute errors between the experimental and calculated *E*_ad_ values range from 0.1 to 0.8 kcal/mol (Table S2), which are within experimental errors (1 kcal/mol) for *E*_ad_ [66]. The *E*_ad_ values obtained from ab initio molecular dynamics for 6 organic molecules onto graphene were also in excellent agreement with experimental values with small errors (0.1~0.9 kcal/mol) [24]. Thus, it can be concluded that the DFT method in this study can give a viable estimation on the *E*_ad_ values.

It is known that macroscopically and empirically determined log*K* is theoretically related with the thermodynamic quantities Δ*G*, enthalpy (Δ*H*), entropy (Δ*S*), temperature (*T*) and universal gas constant (*R*) through the equation [67]:

log*K =* −Δ*G*/2.303*RT* + log*C* = −(Δ*H* − *T*Δ*S*)/2.303*RT* + log*C* (4)

where the slope term −1*/*2.303*RT* can be calculated as −0.73 mol kcal^−1^ at *T* = 300 K. The term log*C* in Equation (4) is a constant of investigated systems consisting of water with a specific volume (*V*, mL) and adsorbent with a specific mass (*m*, g). As detailed in the Supplementary Materials, *C* = *V*/*m* (mL/g). From another point of view, the terms *S*_A_ and *Z* in Equation (3) jointly lead to the constant term of Equation (4).

The Δ*G*_MD_ and log*K*_cal_ values (Table S3) for adsorption of 15 aromatic compounds onto graphene were calculated by the MD integrated with the ABF method. log*K*_cal_ and experimentally determined log*K*_exp_ [20] for the aromatics correlate with Δ*G*_MD_ significantly, respectively:

log*K*_cal_ = −0.70Δ*G*_MD_ − 0.8, *n* = 15, *r* = 0.99, *p* < 0.01 (5)

log*K*_exp_ = −0.68Δ*G*_MD_ + 0.3, *n* = 15, *r* = 0.81, *p* < 0.01 (6)

It can be seen that the slopes in Equations (5) and (6) are close to the theoretical value (−0.73) of Equation (4), indicating Equations (5) and (6) conform to the theoretical relationship of Equation (4). As indicated by Figure 1b, log*K*_exp_ significantly correlates (*r* = 0.84) with the log*K*_cal_ too. Comer et al. [30] also reported that log*K*_cal_ significantly correlated with corresponding experimental values for adsorption of aromatics with a variety of functional groups onto carbon nanotubes (*r* = 0.90). Therefore, it can be inferred that the MD simulation coupled with ABF in this study can give reliable estimations for log*K* or Δ*G*_MD_.

Although the current study indicates the experimental log*K*_exp_ and *E*_ad_ values correlate well with the MD or DFT calculated ones, it deserves mentioning that possible biases due to wrong experimental determinations could not be addressed by the models. Although the DFT or MD methods can estimate the adsorption parameters (*E*_ad_ and log*K*), it can be impractical to calculate the adsorption parameters for all organics owing to the enormous computational cost. Therefore, it is necessary to develop high-throughput prediction models for the adsorption parameters.

### 3.2. G(Z) and E_ad_ of the Adsorbates onto BP

The penalty scores of force field parameters for methyl-2-methylbenzoate, 2,4-dinitrotoluene, malonic acid and 1,2-dinitrobenzene are >10. To improve the accuracy of *G*(*Z*), the force field parameters are further refined by the ffTK [53], and the refined parameters are listed in Tables S4–S10.

**Figure 2:**
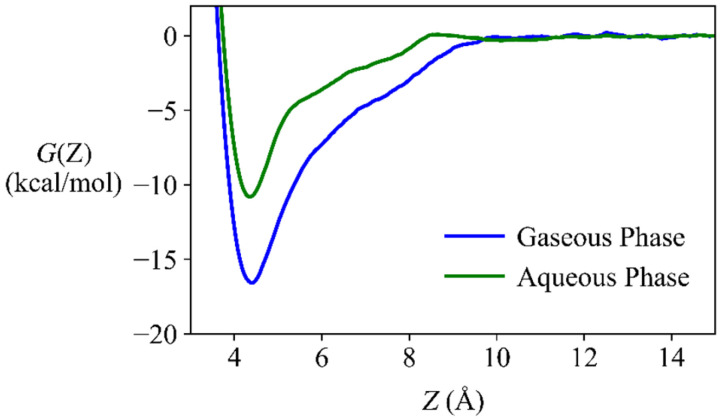
Variation in free energy *G*(*Z*) along a transition coordinate (*Z*) that was defined as the vertical distance between the center of mass for an adsorbate (here taking phenanthrene as an example) and of the BP surface.

Figure 2 (taking phenanthrene as an example) shows that variation in *G*(*Z*) along the adsorption distances exhibits a typical V-shape. It indicates that the adsorption distance of 4.4 Å between the BP surface and phenanthrene is a crest, blow and above which repulsion and attraction become dominant, respectively. *G*(*Z*_min_) of the other adsorbates also occur at about *Z* = 4.4 Å. Azhagiya Singam et al. [26] investigated adsorption thermodynamics of a diverse set of aromatic compounds onto graphene by MD, and found that *Z*_min_ ranged from 3.5 to 3.8 Å.

Δ*G*_MD_ for all the adsorbates ranges from −3.8 to −18.8 kcal/mol in the gaseous phase and from −2.2 to −12.7 kcal/mol in the aqueous phase (Table S11), indicating BP can capture the aromatic and aliphatic compounds spontaneously. The DFT calculated *E*_ad_ values range from −7.5 to −28.7 kcal/mol and −4.7 to −24.7 kcal/mol in gaseous and aqueous phases, respectively (Table S1). The MD calculated log*K* values range from 2.1 to 12.5, and 1.0 to 8.1, in gaseous and aqueous phases, respectively. log*K* correlates with Δ*G*_MD_ or *E*_ad_ as follows:

log*K*_aqueous_ = −0.68Δ*G*_MD(aqueous)_ − 0.7, *n* = 41, *r* = 0.99, *p* < 0.01 (7)

log*K*_gaseous_ = −0.70Δ*G*_MD(gaseous)_ − 0.7, *n* = 41, *r* = 0.99, *p* < 0.01 (8)

log*K*_aqueous_ = −0.32*E*_ad(aqueous)_ − 1.0, *n* = 41, *r* = 0.86, *p* < 0.01 (9)

log*K*_gaseous_ = −0.48*E*_ad(gaseous)_ − 2.1, *n* = 41, *r* = 0.93, *p* < 0.01 (10)

It is under the expectation that the calculated log*K*_aqueous_ or log*K*_gaseous_ correlated with Δ*G*_MD(aqueous)_ or Δ*G*_MD(gaseous)_ significantly, since the *K* values were derived from the MD calculated *G*(*Z*) with Equation (3). Similar to Equations (5) and (6), the slopes of Equations (7) and (8) are close to the theoretical value −0.73 mol kcal^−1^, indicating Equations (7) and (8) also conform to the theoretical relationship of Equation (4).

Besides, since *E*_ad(aqueous)_ or *E*_ad(gaseous)_ are essentially Δ*H* in Equation (4), and they do not carry information on Δ*S* in the adsorption, it is not unexpected that the statistical significance of Equations (9) and (10) as indicated by the *r* values, is inferior to that of Equations (7) and (8).

It deserves mentioning that the intercepts of Equations (7) and (8) are close to that of Equation (5). According to Equation (3), the intercepts are relevant with log*S*_A_. Since the difference (0.04) in theoretical log*S*_A_ values between pristine BP and graphene is negligible, it is rational that the intercepts of Equations (5), (7) and (8) are similar. As detailed in the Supplementary Materials, the theoretical intercept of Equation (4) calculated from the MD simulations is −1.0 (Table S12). The intercepts in Equations (5), (7) and (8) are generally close to the theoretical value.

Nevertheless, the intercepts of Equations (5) and (6) are quite disparate. The disparity may lie in the difference in *S*_A_ between the MD simulation and the experiments. Compared with the theoretical *S*_A_ value of pristine graphene in the MD simulation, the *S*_A_ values in the experiment could not be uniform, let alone the log*K*_exp_ values obtained from different experimental studies [20]. For the same batch of adsorption experiments, the *S*_A_ values of graphene are within a certain distribution range. Thus, it can be inferred that the nonuniform *S*_A_ values in the experiments lead to the intercept of Equation (6).

The Δ*G*_MD_ values in the gaseous phase are 1.6~6.1 kcal/mol lower than those in the aqueous phase (Table S11) and the DFT calculated *E*_ad_ values in the gaseous phase are 1.0~6.9 kcal/mol lower than those in the aqueous phase (Table S1), indicating water plays a negative role in the adsorption. The trajectory of the MD simulations shows that water molecules form a stable hydrogen bond network on the BP surface (Figure S3) and reduce the accessibility of the adsorbates to the BP surface. Previous MD simulations on adsorption of biomolecules onto graphene oxide and TiO_2_ nanomaterials also indicated the competing behavior of water [68,69].

The MD calculation was also performed to examine the binding strength of water onto BP in the gaseous phase. Δ*G*_MD_ of water is −1.7 kcal/mol, which is higher than those (−2.2~−12.7 kcal/mol) of the 41 adsorbates. Therefore, it is thermodynamically favorable for the adsorbate to replace water molecules during adsorption onto the BP surface in the aqueous phase (Figure S4), although the process requires overcoming extra energy barriers. Previous DFT calculations also indicated that water can impede the adsorption of aromatics onto graphene oxide by interacting with the hydroxy and epoxy groups of graphene oxides [32].

### 3.3. Adsorption Configurations

**Figure 3:**
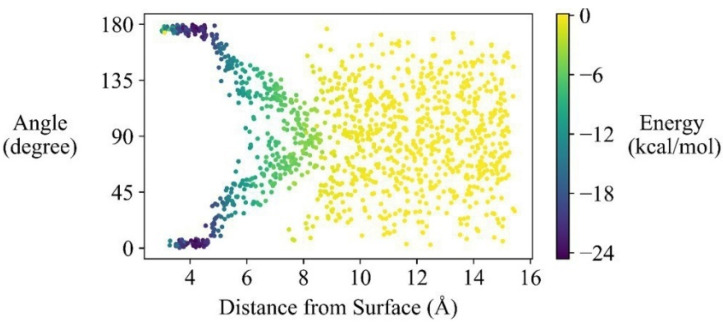
Dihedral angles, distances and interaction energies for configurations of phenanthrene adsorbed onto BP surface (The horizontal ordinates represent vertical distances between phenanthrene and BP, and the vertical ordinates are dihedral angles between the plane formed by the aromatic rings of phenanthrene and BP surface. The color bars represent values of interaction energies including electrostatic and van der Waals interactions. The dark color corresponds to strong adsorption.).

Dihedral angles between the plane formed by the aromatic rings of the adsorbates and the BP surface, vertical distances between the adsorbates and the BP surface, and corresponding interaction energies calculated from MD, are shown in Figure 3, taking phenanthrene as an example. The results for the other 40 adsorbates are shown in Table S13. The dominant adsorption configurations were identified by analyzing the interaction energy of each configuration in the trajectory, as all the accessible orientations of the adsorbates have been sampled during the *G*(*Z*) calculation. Taking phenanthrene as an example, all the configurations sampled for the *G*(*Z*) calculation are shown in Figure S5.

The most energetically favorable adsorption configuration is the adsorbate roughly parallelling to the BP in a distance of around 4.1 Å. The DFT results also show that the adsorbates are roughly parallel to the BP surface at a distance of about 4.2 Å (Table S14). The parallel adsorption configurations were also observed for adsorption of 2,3,7,8-tetrachlorodibenzo-*p*-dioxin, nucleobases and amino acids onto phosphorene [35,36,70].

For adsorption onto graphene, carbon nanotubes and boron nitride nanosheets, DFT or MD simulations also showed that the aromatic rings of adsorbates were nearly parallel to the nanomaterial surface [25,30,41]. Generally, the adsorption of the compounds onto these nanomaterials is mainly physically, which is dominated by van der Waals forces. It can be seen that the parallel configuration can maximize the contact areas between the nanomaterials and the adsorbates, and thus enhance the van der Waals interactions.

Besides, BP was found to deform to different extents after the adsorption. Root mean square displacements (*RMSD*, detailed in Table S15) between the initial and the optimized adsorption geometries of the BP were adopted to describe the deviation/deformation. *RMSD* of BP adsorbing acetaldehyde is 0.01 nm, and the deformation is the slightest. *RMSD* of BP adsorbing pyrene is 0.21 nm, and the deformation is the largest. Acetaldehyde and pyrene correspond to the highest and the lowest values of *E*_ad_ and Δ*G*_MD_ among the 41 compounds, respectively (Tables S1 and S11). Previous studies also revealed that the wrinkles of graphene and graphene oxides can promote their adsorption for organic pollutants [15,32,71].

Pearson coefficients between *RMSD* and *E*_ad_ or Δ*G*_MD_ in gaseous and aqueous phase range from −0.48 to −0.58, indicating that *E*_ad_ or Δ*G*_MD_ decreases with the increase of the deformation. Zhao et al. [6] also found that BP had wrinkles after adsorbing methylene blue or congo red, and the deformation can increase adsorption sites or adsorption capacities.

### 3.4. pp-LFER Models

The constructed pp-LFER models are as follows:

Gaseous phase: log*K* = 0.2 − 0.0076*E* − 1.1*S* − 1.1*A* + 1.4*B* + 1.5*L*

*n*_tra_ = 33, *R*^2^_adj_ = 0.96, *RMSE*_tra_ = 0.35, *Q*^2^_LOO_ = 0.95, *Q*^2^_kfold (*k* = 5, 5000)_ = 0.95,

*n*_ext_ = 8, *RMSE*_ext_ = 0.35, *Q*^2^_ext_ = 0.97 (11)

Aqueous phase: log*K* = −1.7 + 0.65*E* + 0.75*S* + 0.048*A* − 0.095*B* + 4.0*V*

*n*_tra_ = 33, *R*^2^_adj_ = 0.87, *RMSE*_tra_ = 0.46, *Q*^2^_LOO_ = 0.83, *Q*^2^_kfold (*k* = 5, 5000)_ = 0.82,

*n*_ext_ = 8, *RMSE*_ext_ = 0.43, *Q*^2^_ext_ = 0.90 (12)

Gaseous phase: |*E*_ad_| = 6.0 + 1.4*E* − 7.4*S* + 1.2*A* + 6.6*B* + 3.4*L*

*n*_tra_ = 33, *R*^2^_adj_ = 0.97, *RMSE*_tra_ = 0.62, *Q*^2^_LOO_ = 0.96, *Q*^2^_kfold (*k* = 5, 5000)_ = 0.96,

*n*_ext_ = 8, *RMSE*_ext_ = 0.61, *Q*^2^_ext_ = 0.97 (13)

Aqueous phase: *|E*_ad_| = 1.5 + 3.5*E* − 3.8*S* + 0.89*A* + 1.7*B* + 13*V*

*n*_tra_ = 33, *R*^2^_adj_ = 0.96, *RMSE*_tra_ = 0.66, *Q*^2^_LOO_ = 0.95, *Q*^2^_kfold (*k* = 5, 5000)_ = 0.94,

*n*_ext_ = 8, *RMSE*_ext_ = 0.73, *Q*^2^_ext_ = 0.96 (14)

The |*E*_ad_| or log*K* values predicted by the pp-LFER models agree well with the DFT or MD calculation values (Figure S6). Values of the statistical parameters (*R*^2^_adj_, *Q*^2^_LOO_, *Q*^2^_kfold_ and *Q*^2^_ext_) indicate that these models possess high goodness-of-fit, robustness and predictive abilities [72]. As shown by the Williams plots (Figure S7), there are no outliers or high leverage points in the models. Thus, the pp-LFER models can offer an efficient way for high-throughput estimating the adsorption parameters (*E*_ad_ and log*K*) of other aliphatic and aromatic compounds structurally similar to the training set compounds.

Table 1 and Table S16 list the relative contributions of intermolecular interactions to the overall adsorption. It can be seen that the *sS* term contributes negatively (−26%) to |*E*_ad_|, and positively (20%) to log*K*. The contributions of the other terms to |*E*_ad_| are not equal to those to log*K* either. It can be concluded that the relative contributions of a specific interaction to |*E*_ad_| and log*K* are quite disparate. As can be seen from Equation (4), the disparity can be due to the absence of the entropic term (*T*ΔS) in *E*_ad_ of the adsorption.

When the contribution of each pp-LFER term to log*K* is decomposed into the enthalpic (|*E*_ad_|) and entropic (*T*Δ*S*) components, the disparity can be explained explicitly. For example, the contribution of the *sS* term to log*K* is 20%, which is approximately equal to the sum of the enthalpic (−70%) and entropic (93%) contributions. Thus, the contribution of the *sS* term to log*K* is opposite to that to |*E*_ad_|, since the negative enthalpic contribution is exceeded by the positive entropic contribution.

The total enthalpic contribution of the five terms is 243%, which is far greater than that of entropy (−94%). The result indicates that enthalpy dominates the adsorption, which echoes the significant correlations between log*K* and *E*_ad_ as indicated by Equations (9) and (10). Shen et al. [73] also found that the enthalpy dominates the adsorption of nitroaromatics on multiwall carbon nanotubes by experimental studies.

log*K* can also be decomposed into the contribution of water (Δ*G*_H2O_) and of the interaction (Δ*G*_MD(gaseous)_) between BP and the compounds in the gaseous phase. The total contribution of water is −61%, in which *vV* term (−49%) is the most significant factor. The *vV* term of Δ*G*_H2O_ may represent energy required to form a water cavity onto the BP surface to accommodate the adsorbates [33,67]. Thus, it can be concluded that the negative contribution of water to the adsorption mainly arises from the cavity effect.

Previous pp-LFER models on graphene, graphene oxides, carbon nanotubes were also adopted to analyze the contributions of the different interactions [17,19,20,29,74]. However, the previous studies did not distinguish the contributions of enthalpy, entropy and water to log*K*. In the current study, the contributions of entropy, enthalpy and water to the interactions were further estimated by the pp-LFERs integrated with the MD and DFT results.

### 3.5. Comparisons with Graphene

**Figure 4:**
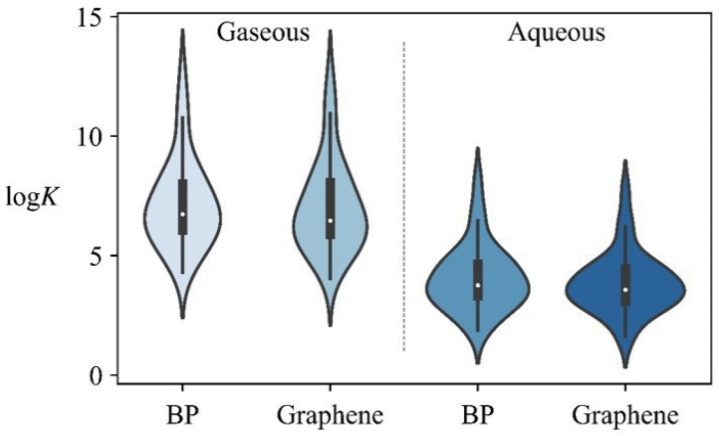
Violin plot of log*K* values on BP and graphene in the gaseous and aqueous phase. The width of the plot represents the frequency of the data points.

Table S17 lists the Δ*G*_MD_ and log*K* values for adsorption of 30 aromatic compounds onto BP and graphene in gaseous and aqueous phases. The log*K* values (Figure 4) for BP range from 1.9 to 12.5, which are close to those (1.6~12.4) for graphene. The Δ*G*_MD_ differences between BP and graphene range from −0.9 to 0.3 kcal/mol in gaseous and aqueous phases. The results indicate that the affinity of the aromatics to BP is comparable to that to graphene. Lazar et al. [28] also found that experimental Δ*H* of benzene adsorption onto BP was equal to that onto graphene.

The contributions of the different interactions to the overall adsorption for the 30 aromatics onto BP and graphene are shown in Figure S8. The contribution of each term to BP is close to that of graphene, explaining why the adsorption affinity of BP to aromatic hydrocarbons was comparable to that of graphene. The models in this study indicated that no matter for BP or graphene, the dispersion interactions (*lL* and *vV*) dominate the adsorption in the gaseous phase and the aqueous phase. Previous pp-LFER models for the adsorption of organics onto different carbon nanomaterials including graphene, carbon nanotubes and graphene oxides (Table 2) also indicated that the dispersion interactions play crucial roles.

Although the number and category of the compounds in the reported models differ from those in this study, the pp-LFER models for predicting *K* values of BP and graphene (0.86~0.97) developed in the current study outperformed or was comparable to the previous ones (0.83~0.93) in terms of *R*^2^_train_. Thus, the pp-LFER models in the current study not only are valuable for understanding the contributions of the different intermolecular interactions but also reliable in predicting log*K* values for estimating adsorption capacities.

## 4. Conclusions

In this study, DFT and MD calculations were combined with pp-LFER models to investigate the adsorption of aromatic chemicals onto BP and unveil differences in adsorption mechanisms between BP and the widely investigated graphene. The results indicate that the DFT and MD simulation can give reliable *E*_ad_, log*K* and Δ*G*_MD_ values. The dispersion interactions dominate the adsorption of the aromatics in the gaseous phase and aqueous phase. As extra energy barriers need to be overcome to form a water cavity onto the BP surface to accommodate the adsorbates, the adsorption in the gaseous phase is more favorable than in the aqueous phase. The affinity of the aromatics to BP is comparable to that of graphene. The pp-LFER models can serve as effective tools to rank adsorption capacities and to estimate adsorption parameters of organics onto BP.

## Figures and Tables

**Table 1:** Relative contributions of intermolecular interactions to the overall adsorption of organic chemicals onto BP in an aqueous phase.

Term	|*E*_ad_|	log*K*	^a^ log*K*		^b^ log*K*	
			*T*Δ*S*	|*E*_ad_|	Δ*G*_H2O_	Δ*G*_MD_ (Gaseous)
*eE*	23%	17%	−47%	62%	−5%	21%
*sS*	−26%	20%	93%	−70%	−8%	30%
*aA*	1%	0	−3%	3%	2%	−2%
*bB*	3%	−1%	−11%	9%	−1%	−1%
*vV*	88%	111%	−126%	239%	−49%	162%
^c^ Sum	89%	147%	−94%	243%	−61%	210%

^a^ Δ*G*_MD(aqueous)_ was decomposed with Equation (4) into *T*Δ*S* and *E*_ad_ using the results of MD and DFT, *T*Δ*S* and *E*_ad_ were further decomposed into various intermolecular interactions by the pp-LFERs. ^b^ Δ*G*_MD(aqueous)_ was decomposed into Δ*G*_H2O_ (Δ*G*_H2O_ = Δ*G*_MD(aqueous)_ – Δ*G*_MD(gaseous)_) and Δ*G*_MD(gaseous)_. Δ*G*_H2O_ and Δ*G*_MD(gaseous)_ were also decomposed into various intermolecular interactions by the pp-LFERs. ^c^ The sum consists of the five terms with the explicit interactions and does not include the contributions of the intercept term in the pp-LFERs. The contribution is average fractions of the terms in the sum of the six terms in the pp-LFERs (detailed in the Supplementary Materials).

**Table 2:** Comparison of pp-LFER models developed in the current and previous studies.

No.	Nanomaterial	Phase	*N*_train_	*R* ^2^_train_	Prediction Model
1	SWCNT [34]	aqueous	30	0.87	log*K* = −1.3 + 0.40*E* + 0.36*S* + 0.93*A* − 3.9*B* + 2.8*V*
2	MWCNT [17]	aqueous	29	0.83	log*K* = −4.3 + 0.61*S* + 0.050*A* − 0.48*B* + 4.5*V*
3	MWCNT [75]	aqueous	28	0.93	log*K* = −1.3 + 0.043*E* + 1.7*S* − 0.37*A* − 2.7*B* + 4.1*V*
4	Graphene [20]	aqueous	29	0.89	log*K* = −1.4 + 0.11*E* + 1.4*S* + 0.42*A* − 3.8*B* + 2.2*V*
5	Graphene [29]	aqueous	35	0.88	log*K* = −1.8*ε*_α_ − 1.2*ε*_β_ + 1.3*q*^+^ − 1.5*q*^−^ + 1.0*V* − 1.6*π* + 42
6	Graphene oxide [20]	aqueous	36	0.84	log*K* = −1.4 + 0.29*E* + 0.28*S* − 0.19*A* − 2.6*B* + 2.6*V*
7	Graphene oxide [74]	aqueous	36	0.92	log*K* = −1.7 + 0.93*E* + 0.060*S* − 0.38*A* − 1.9*B* + 2.2*V*
8	BP (this study)	aqueous	33	0.87	log*K* = −1.7 + 0.65*E* + 0.75*S* + 0.048*A* – 0.095*B* + 4.0*V*
9	BP (this study)	gaseous	33	0.96	log*K* = 0.2 − 0.0076*E* − 1.1*S* − 1.1*A* + 1.4*B* + 1.5*L*
10	Graphene (this study)	aqueous	30	0.86	log*K* = −1.6 + 0.42*E* + 1.0*S* + 0.26*A* − 0.78*B* + 4.0*V*
11	Graphene (this study)	gaseous	30	0.97	log*K* = 0.052 − 0.043*E* − 0.62*S* − 0.78*A* − 0.36*B* + 1.5*L*

Multi-walled carbon nanotubes (MWCNT); single-walled carbon nanotubes (SWCNT); black phosphorus (BP); *N*_train_: the number of compounds used for developing the model; *R*^2^_train_: determination coefficient of training sets.

## Data Availability

The data presented in this study are available in the supplementary material of this article.

## Supplementary Materials

The following supporting information can be downloaded at: https://www.mdpi.com/article/10.3390/nano12040590/s1, Figures S1–S5: Computational details and trajectory analysis of MD simulations; Figures S6–S8: Correlations between predicted and calculated values; Williams plots; Average contributions of different interactions to the overall adsorption; Tables S1–S3, S11 and S17: MD or DFT calculated log*K*, Δ*G*_MD_ and *E*_ad_ values for graphene or BP; Tables S4–S10: Refined force field parameters; Tables S13–S14: Adsorption configurations for DFT and MD calculations. Tables S12, S15 and S16: Theoretical intercept values, *RMSD* values and relative contributions of different interactions to the overall adsorption of BP in the gaseous phase. The supporting information also consists of other computational details and derivation processes mentioned in the main text.
