# Supplementary material for: Simulating and Predicting Adsorption of Organic Pollutants onto Black Phosphorus Nanomaterials
Source: Nanomaterials (Basel). 2022 Feb 9;12(4):590. doi: 10.3390/nano12040590 (PMC8875661; doi:10.3390/nano12040590)
Supplement: Supplementary file 1 [file nanomaterials-12-00590-s001.zip › nanomaterials-1564605-supplementary.pdf]

Supporting Information

Simulating and Predicting Adsorption of Organic Pollutants onto Black Phosphorus Nanomaterials

Lihao Su [1], Ya Wang [1], Zhongyu Wang [1], Siyu Zhang [2,*], Zijun Xiao [1], Deming Xia [1] and Jingwen Chen [1,*]

[1] Key Laboratory of Industrial Ecology and Environmental Engineering (Ministry of Education), Dalian Key Laboratory on Chemicals Risk Control and Pollution Prevention Technology, School of Environmental Science and Technology, Dalian University of Technology, Dalian 116024, China; su_lihao@mail.dlut.edu.cn (L.S.); yaanne@mail.tsinghua.edu.cn (Y.W.); wzy1989@mail.dlut.edu.cn (Z.W.); xzj960729@mail.dlut.edu.cn (Z.X.); xiadm@mail.dlut.edu.cn (D.X.)

[2] Key Laboratory of Pollution Ecology and Environmental Engineering, Institute of Applied Ecology, Chinese Academy of Sciences, Shenyang 110016, China

* Correspondence: syzhang@iae.ac.cn (S.Z.); jwchen@dlut.edu.cn (J.C.)

This supporting information (SI) file consists of 46 pages, including 8 figures and 17 tables.

Contents

Structural information about graphene model for density functional theory (DFT) and molecular dynamics (MD) calculations.......... 3

Convergence of potential mean force (PMF) curves/free energy [*G*(*Z*)] 3

Theory description on relationships between equilibrium constant (*K*) and free energy change (Δ*G*) 4

Contributions of entropy, enthalpy and water to log*K*.......... 5

pp-LFER models for log*K* of BP and graphene. 7

Figure S1. Construction of adsorption system for MD simulation. 8

Figure S2. Convergence analysis of free energy [*G*(*Z*)].......... 8

Figure S3. Water molecules onto BP surface 9

Figure S4. Representative trajectory snapshots of the adsorption of phenanthrene onto BP 9

Figure S5. Superimposed conformations of phenanthrene sampled in free energy calculations........ 10

Figure S6. Plots of the pp-LFER models predicted $|E_{ad(pre)}|$ or logK_{pre} *versus* DFT or MD calculated $|E_{ad(cal)}|$ or logK_{cal} in the gaseous and aqueous phases........ 10

Figure S7. Williams plots of standardized residuals (δ^*) *versus* leverage (h) for the pp-LFER models on $|E_{ad}|$ or log$K$ in the gaseous and aqueous phases........ 11

Figure S8. Average contributions of each term of pp-LFER to the overall adsorption (log$K$) of BP and graphene........ 11

Table S1. MD calculated log$K$ and DFT calculated $|E_{ad}|$ values for the adsorption of 41 compounds onto BP in aqueous and gaseous phases........ 12

Table S2. Comparison of experimental and DFT calculated E_{ad} values for adsorption of organic compounds onto graphene in gaseous phase........ 15

Table S3. Comparison of experimental and MD calculated values for adsorption of aromatic compounds onto graphene in aqueous phase........ 15

Table S4. Refined charge parameters for methyl-2-methylbenzoate........ 16

Table S5. Refined dihedral parameters for methyl-2-methylbenzoate........ 17

Table S6. Refined charge parameters for 2,4-dinitrotoluene........ 17

Table S7. Refined dihedral parameters for 2,4-dinitrotoluene........ 19

Table S8. Refined angle parameters for malonic acid........ 19

Table S9. Refined dihedral parameters for malonic acid........ 19

Table S10. Refined dihedral parameters for 1,2-dinitrobenzene........ 20

Table S11. Adsorption free energy (ΔG_{MD}) of 41 compounds onto BP in aqueous and gaseous phases........ 20

Table S12. Theoretical intercept values (logC) in linear relationships between the MD calculated logK_{cal} and ΔG_{MD} for graphene........ 22

Table S13. Analysis of the conformations of the adsorbates onto BP surface........ 23

Table S14. Equilibrium configurations of the adsorbates onto BP surface........ 32

Table S15. Root mean square deviation (*RMSD*) between the initial and the optimized adsorption configurations of BP........ 40

Table S16. Relative contributions of different interactions to the overall adsorption of BP in the gaseous phase........ 42

Table S17. Adsorption free energy (ΔG_{MD}) and log$K$ values for 30 aromatic compounds onto BP and graphene in aqueous and gaseous phases. 43
REFERENCES 45

Structural information about graphene model for density functional theory (DFT) and molecular dynamics (MD) calculations

A graphene supercell (8 × 5 × 1) with 160 carbon atoms was built for the DFT calculations. A periodic box of 19.65 × 21.27 × 25.00 Å^3 was applied in the adsorbate-graphene system.

A three-layer graphene adsorbent in a periodic box of 36.00 × 36.00 × 40.00 Å^3 was built for the MD simulations. The generic atomic type for aromatic carbon (CG2R61) archived in CHARMM General Force Field (CGenFF) [1] was adopted to represent graphene carbon atoms, which was successfully employed to simulate adsorption of compounds onto graphene [2]. Other parameters for the MD and DFT calculations on the graphene system were the same as those for the calculations on black phosphorus (BP), which were given in the main text.

Convergence of potential mean force (PMF) curves/free energy [$G(Z)$]

The convergence of $G(Z)$ of each simulation was checked. Taking phenanthrene as an example, curves of $G(Z)$ sampled at 50 and 100 ns almost overlapped (Figure S2a), and the normalized $G(Z)$ of 0 ~ 50 ns was almost equal to that of 50 ~ 100 ns (Figure S2b). Thus, a simulation time of 100 ns is sufficient for the convergence of the $G(Z)$. Likewise, the $G(Z)$ convergence of other compounds was checked.

Theory description on relationships between equilibrium constant (*K*) and free energy change (Δ*G*)

K for the partitioning of a given compound between two phases 1 and 2 is related to their corresponding Δ*G*, enthalpy (Δ*H*), and entropy (Δ*S*):

$$K = x_1/x_2 = e^{-\Delta G/RT} = e^{-(\Delta H - T\Delta S)/RT} \quad (1)$$

where R is the universal gas constant (8.314 J mol^{-1} K^{-1}), T is temperature, and x_i is the molar fraction of the compound in phase i. K from adsorption experiments, is not dimensionless, and is expressed as:

$$K = c_1/c_2 = (x_1/x_2) \times (V/m) = (V/m) \times e^{-\Delta G/RT} = (V/m) \times e^{-(\Delta H - T\Delta S)/RT} \quad (2)$$

where c_1 is the concentration (mol g^{-1}) of compound onto adsorbents (phases 1), c_2 is the concentration (mol mL^{-1}) of compound in solutions (phases 2), V (mL) is the volume of solutions for the investigated systems, m (g) is the mass of the adsorbent. Eq. (2) can be converted into:

$$\log K = -\Delta G/2.303RT + \log C = -(\Delta H - T\Delta S)/2.303RT + \log C \quad (3)$$

At infinite dilution, the factor (C) is a constant, which is related to V and m (C = V/m).

Theoretically, there is also an intrinsic correlation between the MD-calculated $\log K_{cal}$ and ΔG_{MD}:

$$\log K_{cal} = -\Delta G_{MD}/2.303RT + \log C \quad (4)$$

where the slope term $-1/2.303RT$ is -0.73 mol kcal^{-1} at T = 300 K. According to eq. (4), the $\log C$ values for the adsorption of 15 aromatics onto graphene were calculated (Table S12). Mean of the $\log C$ values is -1.0, and standard deviation is 0.1. For the adsorption of 41 adsorbates onto BP, $\log C$ is also equal to -1.0 ± 0.1 no matter in aqueous or gaseous phases.

According to eq. (3) in the main text, $\log C$ is relevant with $\log S_A$ (S_A: specific surface areas of adsorbents). Since the difference (0.04) in theoretical $\log S_A$ values

between pristine BP and graphene is negligible, it is rational that the mean value of log*C* for graphene is equal to that of BP. Thus, it can be inferred that theoretical value of the constant term log*C* for pristine graphene and BP is -1.0.

Contributions of entropy, enthalpy and water to log*K*

According to eq. (3), the contribution of each polyparameter linear free energy relationship (pp-LFER) term to log*K* can be decomposed into the enthalpic and entropic components by pp-LFERs integrated with the MD and DFT results.

For the 33 compounds in the training set, their $\log K_{\mathrm{gaseous}}$ values correlate with ΔG_{MD} in gaseous phase:

$$\log K_{\mathrm{gaseous}} = -0.70\Delta G_{\mathrm{MD}} - 0.7,\ n = 33,\ r = 0.99,\ p < 0.01 \quad (5)$$

Since DFT calculated E_{ad} is essentially ΔH in eq. (3), the above equation is equivalent to:

$$\log K_{\mathrm{gaseous}} = 0.70|\Delta G_{\mathrm{MD}}| - 0.7 = 0.70|E_{\mathrm{ad}}| + 0.70T\Delta S - 0.7 \quad (6)$$

The $|E_{\mathrm{ad}}|$ and $T\Delta S$ terms in eq. (6) can be substituted with their pp-LFERs:

$$\log K_{\mathrm{gaseous}} = 0.70|E_{\mathrm{ad}}| + 0.70T\Delta S - 0.7$$
$$= 0.70 \times (6.0 + 1.4E - 7.4S + 1.2A + 6.6B + 3.4L) + 0.70 \times (-4.7 - 1.5E + 5.7S - 2.6A - 4.8B - 1.2L) - 0.7$$
$$= 0.2 + (0.97E - 5.2S + 0.84A + 4.6B + 2.4L) + (-1.0E + 4.0S - 1.8A - 3.3B - 0.84L) \quad (7)$$

Likewise, for the adsorption of the 33 compounds onto BP in aqueous phase:

$$\log K_{\mathrm{aqueous}} = 0.68|\Delta G_{\mathrm{MD}}| - 0.6$$
$$= 0.68|E_{\mathrm{ad}}| + 0.68T\Delta S - 0.6$$
$$= 0.68 \times (1.5 + 3.5E - 3.8S + 0.89A + 1.7B + 13V) + 0.68 \times (-3.1 - 2.6E + 5.0S - 0.76A - 2.0B - 6.6V) - 0.6$$
$$= -1.7 + (2.4E - 2.6S + 0.60A + 1.1B + 8.8V) + (-1.8E + 3.4S - 0.51A - 1.4B -$$

$4.5V)$ (8)

According to eqs. (7) and (8), the contribution of each interaction term (eE, sS, aA, bB, vV or lL) to log$K$ can be decomposed into the enthalpic ($|E_{ad}|$) and entropic ($T\Delta S$) components. For example, the enthalpic contribution ($P_{eE,\text{ enthalpic}}$) of the eE term to logK_{aqueous} in eq. (8) can be estimated as:

$$P_{eE,\text{ enthalpic}} = \frac{(\sum_{i=1}^{n} e_h E_i)}{\sum_{i=1}^{n}[(e_h + e_s)E_i + (s_h + s_s)S_i + (a_h + a_s)A_i + (b_h + b_s)B_i + (v_h + v_s)V_i + nc]} \quad (9)$$

where e_hE_i, s_hS_i, a_hA_i, b_hB_i and v_hV_i represent enthalpic components of five interaction terms for compound i, which correspond to $2.4E$, $-2.6S$, $0.60A$, $1.1B$ and $8.8V$ in eq. (8), respectively; e_sE_i, s_sS_i, a_sA_i, b_sB_i and v_sV_i represent entropic components of five interaction terms, which correspond to $-1.8E$, $3.4S$, $-0.51A$, $-1.4B$ and $-4.5V$ in eq. (8), respectively; n represents the number of the compounds; c is intercept term, which corresponds to -1.7 in eq. (8).

The contribution of each pp-LFER term to logK_{aqueous} also can be decomposed into contribution of water [$\Delta G_{H2O} = \Delta G_{MD(aqueous)} - \Delta G_{MD(gaseous)}$] and of the interaction [$\Delta G_{MD(gaseous)}$] between BP and the compounds in gaseous phase by pp-LFERs of $\Delta G_{MD(gaseous)}$ and ΔG_{H2O}.

$$\begin{aligned}\log K_{\text{aqueous}} &= 0.68|\Delta G_{MD(aqueous)}| - 0.6\\ &= 0.68|\Delta G_{MD(gaseous)}| - 0.68\Delta G_{H2O} - 0.6\\ &= 0.68 \times (-0.84 + 1.2E + 1.6S - 0.62A - 0.17B + 8.5V) - 0.68 \times (0.7 + 0.30E + 0.42S - 0.75A + 0.19B + 2.6V) - 0.6\\ &= (0.81E + 1.1S - 0.42A - 0.12B + 5.8V) + (-0.21E - 0.28S + 0.51A - 0.13B - 1.7V) - 1.6 \quad (10)\end{aligned}$$

For example, the water contribution ($P_{eE,\text{ H2O}}$) of eE term to log$K$ in eq. (10) can be estimated as:

$$P_{eE,\mathrm{H2O}} = \frac{\left(\sum_{i=1}^{n} e_{\mathrm{H2O}} E_i\right)}{\sum_{i=1}^{n}[(e_{\mathrm{H2O}}+e_{\mathrm{g}})E_i+(s_{\mathrm{H2O}}+s_{\mathrm{g}})S_i+(a_{\mathrm{H2O}}+a_{\mathrm{g}})A_i+(b_{\mathrm{H2O}}+b_{\mathrm{g}})B_i+(v_{\mathrm{H2O}}+v_{\mathrm{g}})V_i+nc]} \quad (11)$$

where $e_{\mathrm{H2O}}E_i$, $s_{\mathrm{H2O}}S_i$, $a_{\mathrm{H2O}}A_i$, $b_{\mathrm{H2O}}B_i$ and $v_{\mathrm{H2O}}V_i$ represent water components of five interaction terms for compound *i*, which correspond to -0.21*E*, -0.28*S*, 0.51*A*, -0.13*B* and -1.7*V* in eq. (10), respectively; $e_{\mathrm{g}}E_i$, $s_{\mathrm{g}}S_i$, $a_{\mathrm{g}}A_i$, $b_{\mathrm{g}}B_i$ and $v_{\mathrm{g}}V_i$ represent the interaction in gaseous phase of the five terms for compound *i*, which correspond to 0.81*E*, 1.1*S*, -0.42*A*, -0.12*B* and 5.8*V* in eq. (10), respectively; *n* represents the number of the compounds; *c* is intercept term, which corresponds to -1.6 in eq. (10).

pp-LFER models for log*K* of BP and graphene.

pp-LFER models on log*K* of BP and graphene in gaseous and aqueous phases are as follows:

Gaseous phase, BP:

$$\log K = 0.2 - 0.38E - 0.44S - 0.63A - 0.12B + 1.6L \quad (12)$$

$n = 30$, $R^2_{\mathrm{adj}} = 0.97$, $RMSE = 0.28$, $Q^2_{\mathrm{LOO}} = 0.96$, $Q^2_{\mathrm{kfold}\,(k=5,\,5000)} = 0.96$

Aqueous phase, BP:

$$\log K = -1.8 + 0.37E + 1.4S + 0.20A - 1.2B + 4.2V \quad (13)$$

$n = 30$, $R^2_{\mathrm{adj}} = 0.87$, $RMSE = 0.45$, $Q^2_{\mathrm{LOO}} = 0.83$, $Q^2_{\mathrm{kfold}\,(k=5,\,5000)} = 0.82$

Gaseous phase, Graphene:

$$\log K = 0.052 - 0.043E - 0.62S - 0.78A - 0.36B + 1.5L \quad (14)$$

$n = 30$, $R^2_{\mathrm{adj}} = 0.97$, $RMSE = 0.31$, $Q^2_{\mathrm{LOO}} = 0.96$, $Q^2_{\mathrm{kfold}\,(k=5,\,5000)} = 0.95$

Aqueous phase, Graphene:

$$\log K = -1.6 + 0.42E + 1.0S + 0.26A - 0.78B + 4.0V \quad (15)$$

$n = 30$, $R^2_{\mathrm{adj}} = 0.86$, $RMSE = 0.43$, $Q^2_{\mathrm{LOO}} = 0.80$, $Q^2_{\mathrm{kfold}\,(k=5,\,5000)} = 0.79$

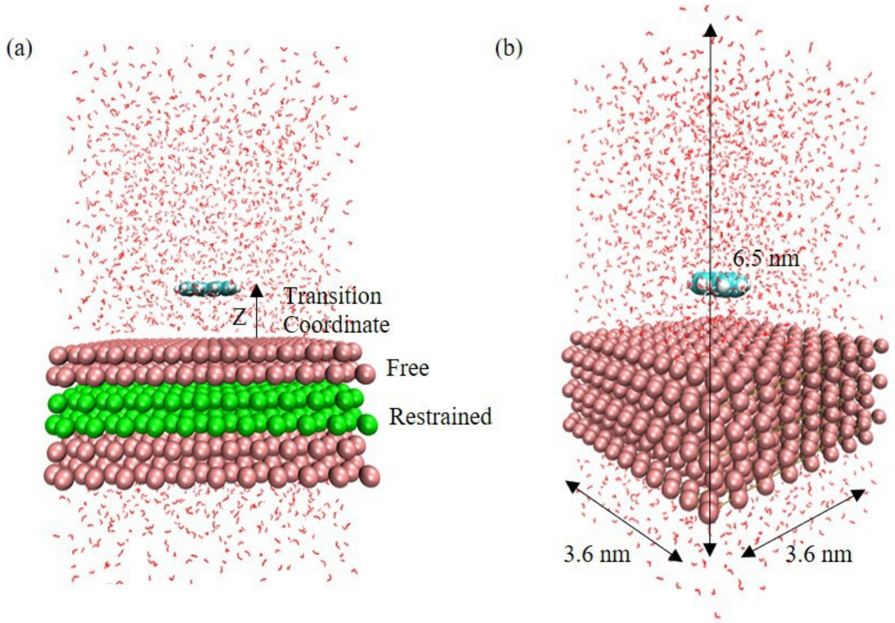

Figure S1. Construction of adsorption system for MD simulation. (**a**) System setup for performing the free energy calculation. (**b**) Initial configuration of the compound-BP adsorption system in aqueous phase.

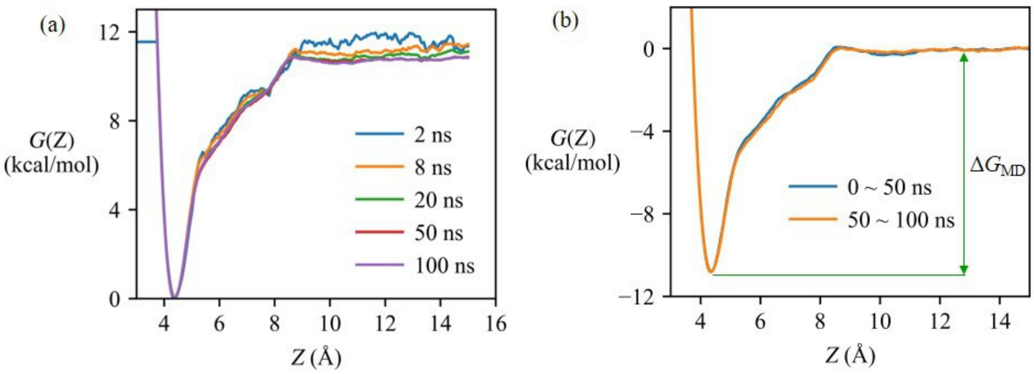

Figure S2. Convergence analysis of free energy [$G(Z)$]. (**a**) Curves of $G(Z)$ sampled at 2 ~ 100 ns. (**b**) A comparison of normalized $G(Z)$ sampled at 0 ~ 50 and 50 ~ 100 ns (ΔG_{MD} is the difference in G between that at position of the minimum G and that at far from the nanomaterial surface.)

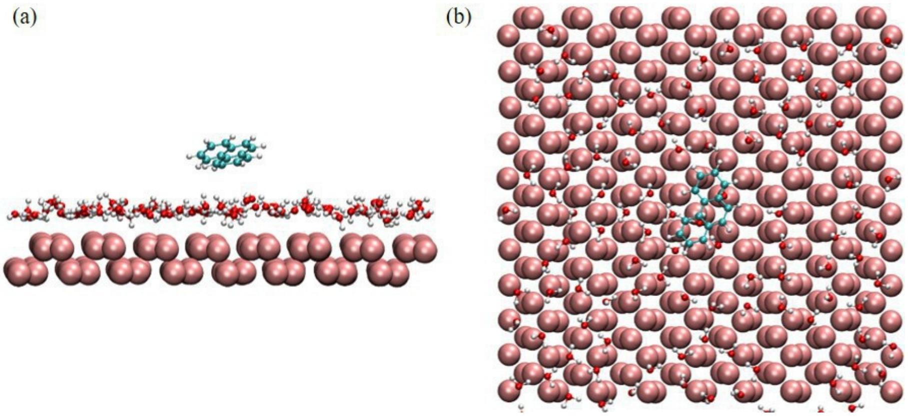

Figure S3. Water molecules onto BP surface (**a**: side view; **b**: top view; pink balls: P atoms; green balls: C atoms; red balls: O atoms; white balls: H atoms.)

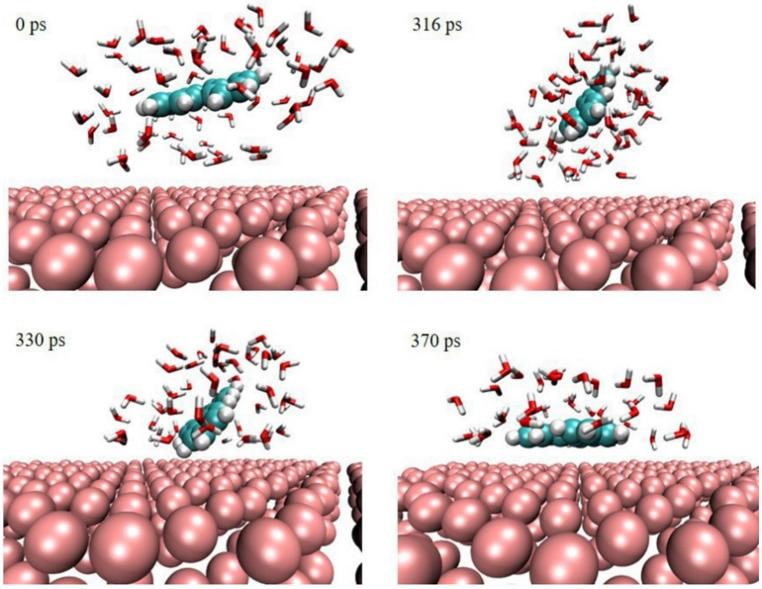

Figure S4. Representative trajectory snapshots of the adsorption of phenanthrene onto BP (For clarity, only water molecules within 5 Å of phenanthrene were shown.)

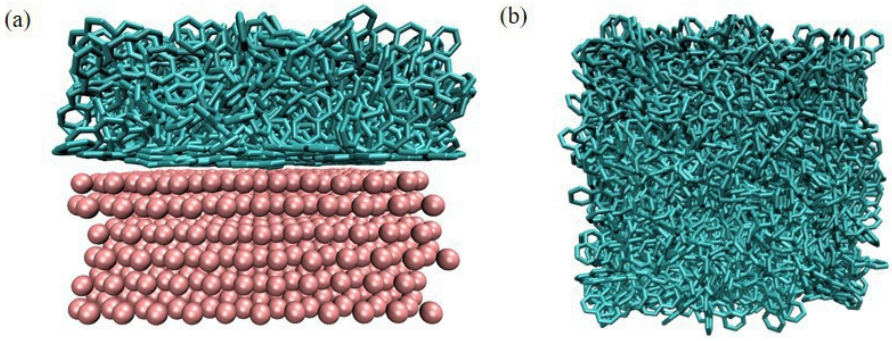

Figure S5. Superimposed conformations of phenanthrene sampled in free energy calculations (**a**: side view; **b**: top view; pink balls: P atoms; green bars: phenanthrene.)

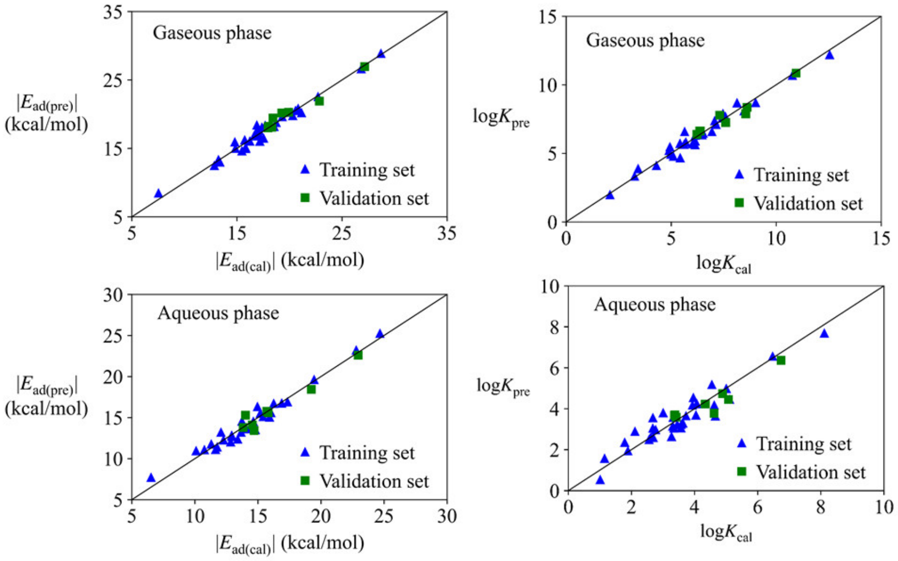

Figure S6. Plots of the pp-LFER models predicted $|E_{\mathrm{ad(pre)}}|$ or logK_{pre} *versus* DFT or MD calculated $|E_{\mathrm{ad(cal)}}|$ or logK_{cal} in the gaseous and aqueous phases.

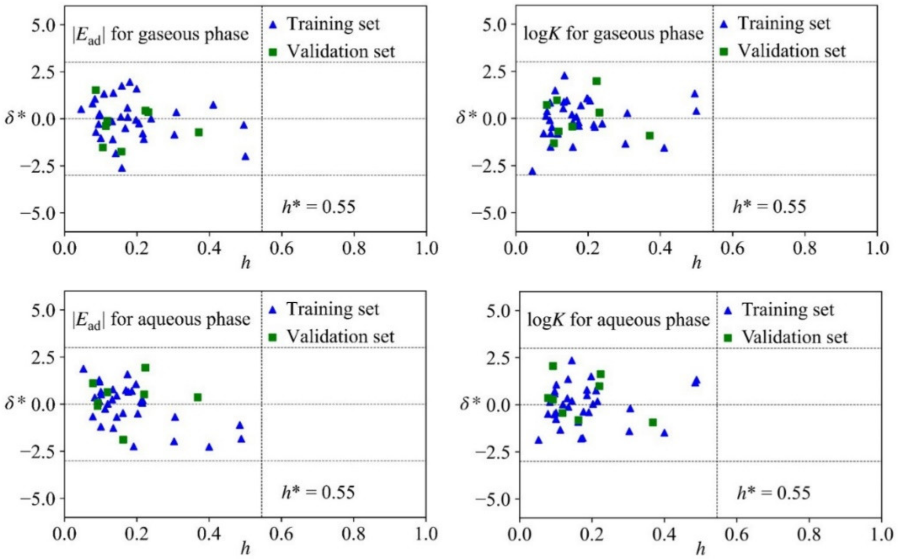

Figure S7. Williams plots of standardized residuals (δ^*) *versus* leverage (h) for the pp-LFER models on $|E_{ad}|$ or log$K$ in the gaseous and aqueous phases.

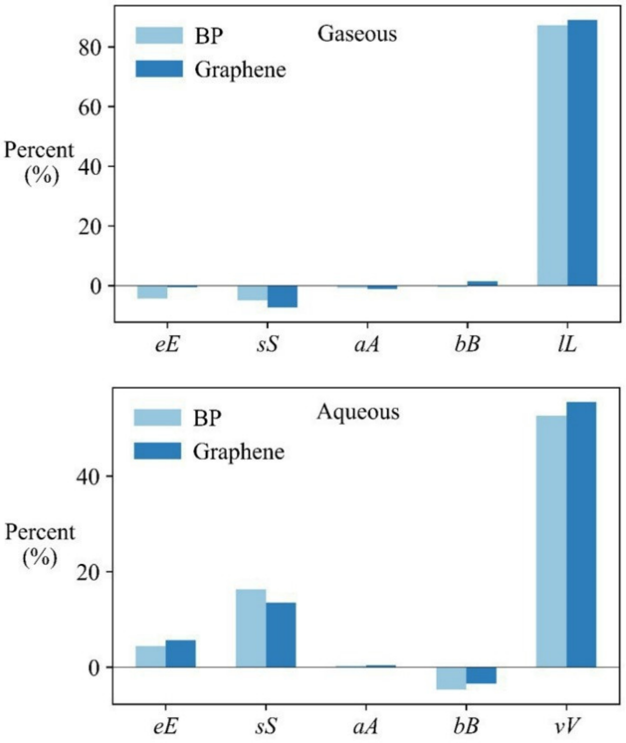

Figure S8. Average contributions of each term of pp-LFER to the overall adsorption (log$K$) of BP and graphene.

Table S1. MD calculated log$K$ and DFT calculated $|E_{ad}|$ values for the adsorption of 41 compounds onto BP in the aqueous and gaseous phases.

Name	Gaseous		Aqueous		[c] Group
	[a] $\|E_{ad}\|$	[b] log$K$	$\|E_{ad}\|$	log$K$	
ethylbenzene	16.6	5.6	13.7	2.7	T
benzonitrile	15.5	6.1	11.8	3.6	T
propylbenzene	16.9	6.3	15.0	3.4	T
p-xylene	17.1	6.1	14.6	3.3	T
4-chlorotoluene	17.2	6.1	14.1	3.4	T
4-chlorophenylamine	18.5	5.6	14.8	2.7	T
m-cresol	17.4	6.0	13.8	3.3	T
3-chlorophenol	16.8	5.7	12.9	3.6	T
3,5-dimethylphenol	21.1	6.9	16.0	4.0	T
bromobenzene	16.2	5.4	13.7	2.8	T
toluene	15.8	5.0	12.9	2.6	T
chlorobenzene	14.9	4.9	12.3	2.6	T
phenol	14.8	5.1	11.3	2.7	T

anthracene	26.8	10.8	22.8	6.5	T
2,4-dinitrotoluene	20.1	9.0	15.2	5.0	T
phenyl acetate	17.1	6.5	14.4	3.0	T
pyrene	28.7	12.5	24.7	8.1	T
malonic acid	13.4	3.4	6.5	1.8	T
4-fluorophenol	15.8	5.4	11.6	3.3	T
1,2-dinitrobenzene	17.1	7.5	12.1	4.0	T
3-bromophenol	17.7	6.1	13.8	3.7	T
iodobenzene	16.9	6.2	15.9	3.3	T
aniline	17.5	4.9	13.4	2.1	T
benzene	13.2	4.3	10.1	1.9	T
isoprene	12.9	3.3	10.8	1.1	T
methyl-2-methylbenzoate	20.6	7.4	17.3	4.1	T
naphthalene	20.3	7.1	16.2	3.9	T
biphenyl	22.7	8.1	19.4	4.5	T
methylbenzoate	19.3	7.1	15.4	4.7	T

ethylbenzoate	20.8	7.6	16.9	4.6	T
nitrobenzene	15.7	6.5	12.8	3.6	T
acetaldehyde	7.5	2.1	4.7	1.0	T
1,3-dinitrobenzene	18.7	8.5	14.3	5.1	T
acetophenone	18.0	6.4	14.6	3.4	V
4-nitrotoluene	18.0	7.6	13.8	4.6	V
1-methylnaphthalene	22.8	8.6	19.2	4.9	V
4-chloroacetophenone	19.3	7.3	15.7	4.3	V
4-ethylphenol	18.4	6.2	14.0	3.4	V
(3-methylphenyl) methanol	19.9	6.4	15.8	3.4	V
phenanthrene	27.1	11.0	22.9	6.7	V
1,4-dinitrobenzene	18.3	8.5	14.7	5.1	V

[a] $|E_{ad}|$ is an absolute value of the adsorption energy E_{ad} (kcal/mol); [b] log$K$ (K, mL/g) is a logarithm of the adsorption equilibrium constant; [c] T and V represent training set and validation set, respectively.

Table S2. Comparison of experimental and DFT calculated E_{ad} values for adsorption of organic compounds onto graphene in the gaseous phase.

Name	[a] Experimental E_{ad} (kcal/mol)	Calculated E_{ad} (kcal/mol)	Absolute Error
Acetonitrile	-7.6 ± 0.3	-7.0	0.6
Benzene	-11.9 ± 0.3	-12.0	0.1
1,4-Dioxane	-10.8 ± 0.1	-10.7	0.1
ethanol	-7.3 ± 0.7	-8.1	0.8
toluene	-13.8 ± 0.4	-14.6	0.8

[a] The experimental E_{ad} values are from other literature [3,4].

Table S3. Comparison of experimental and MD calculated values for adsorption of aromatic compounds onto graphene in the aqueous phase.

Name	[a] logK_{exp}	[b] ΔG_{MD}	[c] logK_{cal}
nitrobenzene	4.0	-6.3	3.6
naphthalene	4.9	-6.8	3.9
1,3-nitrotoluene	4.7	-7.1	4.1
1-naphthylamine	4.8	-6.9	4.0
2-naphthol	4.5	-8.0	4.8
1,3-dinitrobenzene	4.7	-7.8	4.6
phenanthrene	5.5	-10.0	6.2

2-chlorobiphenyl	5.8	-7.4	4.5
3-chlorobiphenyl	6.4	-8.7	5.3
2,4'-dichlorobiphenyl	6.0	-7.7	4.7
bisphenol A	4.6	-6.4	3.8
2,2',5,5'-tetrachlorobiphenyl	7.1	-7.5	4.5
2,3',5,5'-tetrachlorobiphenyl	7.5	-9.1	5.7
2,3,3',4,4',5-hexachlorobiphenyl	8.8	-10.0	6.4
3,3',4,4',5,5'-hexachlorobiphenyl	9.6	-14.1	9.1

[a] The experimental logK_{exp} values are from other literature [5]. [b,c] The adsorption free energy (ΔG_{MD}, kcal/mol) and logK_{cal} (K, mL/g) of the aromatic compounds onto graphene were calculated by MD integrated with an adaptive biasing force (ABF) method in this study.

Table S4. Refined charge parameters for methyl-2-methylbenzoate.

Atom Name	CGenFF Atom Type	Orginal Partial Charge (e)	Refined Partial Charge (e)
O1	OG302	-0.306	-0.306
O2	OG2D1	-0.482	-0.482
C1	CG2R61	0.004	0.282
C2	CG2R61	0.080	-0.453
C3	CG2R61	-0.116	-0.116
C4	CG2R61	-0.110	-0.110
C5	CG331	-0.266	-0.358
C6	CG2R61	-0.115	-0.115
C7	CG2R61	-0.115	-0.115

C8	CG2O2	0.462	0.809
C9	CG331	-0.036	-0.036
H1	HGR61	0.115	0.115
H2	HGR61	0.115	0.115
H3	HGA3	0.090	0.090
H4	HGA3	0.090	0.090
H5	HGA3	0.090	0.090
H6	HGR61	0.115	0.115
H7	HGR61	0.115	0.115
H8	HGA3	0.090	0.090
H9	HGA3	0.090	0.090
H10	HGA3	0.090	0.090

The Force Field Toolkit (ffTK) [6] was employed to further refine the CHARMM-compatible parameters.

Table S5. Refined dihedral parameters for methyl-2-methylbenzoate.

	Atom1	Atom2	Atom3	Atom4	*n*	K_{φ} (kcal/mol)	δ (degrees)
Original Parameters	CG2O2	CG2R61	CG2R61	CG331	2	2.4000	180.00
Refined Parameters	CG2O2	CG2R61	CG2R61	CG331	2	3.0000	180.00

The Force Field Toolkit (ffTK) [6] was employed to further refine the CHARMM-compatible parameters.

Table S6. Refined charge parameters for 2,4-dinitrotoluene.

Atom Name	CGenFF Atom	Orginal Partial	Refined Partial

	Type	Charge (e)	Charge (e)
O1	OG2N1	0.000	-0.340
O2	OG2N1	0.000	-0.340
O3	OG2N1	0.000	-0.340
O4	OG2N1	0.000	-0.340
N1	NG2O1	14.898	0.384
N2	NG2O1	0.000	0.397
C1	CG2R61	13.121	-0.033
C2	CG2R61	15.880	0.347
C3	CG2R61	0.000	-0.249
C4	CG2R61	0.000	0.325
C5	CG2R61	0.000	-0.112
C6	CG2R61	0.000	-0.182
C7	CG331	1.500	-0.268
H1	HGR61	0.000	0.205
H2	HGR61	0.000	0.115
H3	HGR61	0.000	0.160
H4	HGA3	0.000	0.090
H5	HGA3	0.000	0.090
H6	HGA3	0.000	0.090

The Force Field Toolkit (ffTK) [6] was employed to further refine the CHARMM-compatible parameters.

Table S7. Refined dihedral parameters for 2,4-dinitrotoluene.

	Atom1	Atom2	Atom3	Atom4	n	K_{φ} (kcal/mol)	δ (degrees)
Original Parameters	CG331	CG2R61	CG2R61	NG2O1	2	2.4000	180.00
Refined Parameters	CG331	CG2R61	CG2R61	NG2O1	2	3.0000	180.00

The Force Field Toolkit (ffTK) [6] was employed to further refine the CHARMM-compatible parameters.

Table S8. Refined angle parameters for malonic acid.

	Atom1	Atom2	Atom3	K_{θ} (kcal/mol/rad^2)	θ_0 (degree)
Original Parameters	CG2O2	CG321	CG2O2	51.800	107.500
Refined Parameters	CG2O2	CG321	CG2O2	94.930	125.229

The Force Field Toolkit (ffTK) [6] was employed to further refine the CHARMM-compatible parameters.

Table S9. Refined dihedral parameters for malonic acid.

	Atom1	Atom2	Atom3	Atom4	n	K_{φ} (kcal/mol)	δ (degrees)
Original Parameters	OG2D1	CG2O2	CG321	CG2O2	1	0.0000	0.0000
Original Parameters	OG311	CG2O2	CG321	CG2O2	1	0.0000	0.0000

Refined Parameters	OG2D1	CG2O2	CG321	CG2O2	1	0.4450	0.0000
Refined Parameters	OG311	CG2O2	CG321	CG2O2	1	0.5710	0.0000

The Force Field Toolkit (ffTK) [6] was employed to further refine the CHARMM-compatible parameters.

Table S10. Refined dihedral parameters for 1,2-dinitrobenzene.

	Atom1	Atom2	Atom3	Atom4	*n*	K_φ (kcal/mol)	δ (degrees)
Original Parameters	NG2O1	CG2R61	CG2R61	NG2O1	2	5.0000	180.00
Refined Parameters	NG2O1	CG2R61	CG2R61	NG2O1	2	3.0000	180.00

The Force Field Toolkit (ffTK) [6] was employed to further refine the CHARMM-compatible parameters.

Table S11. Adsorption free energy (ΔG_{MD}) of 41 compounds onto BP in the aqueous and gaseous phases.

CAS no.	Name	Gaseous ΔG_{MD} (kcal/mol)	Aqueous ΔG_{MD} (kcal/mol)
100-41-4	ethylbenzene	-9.0	-4.9
100-47-0	benzonitrile	-9.7	-6.3
103-65-1	propylbenzene	-9.9	-5.8
106-42-3	p-xylene	-9.6	-6.0
106-43-4	4-chlorotoluene	-9.8	-6.1

106-47-8	4-chlorophenylamine	-9.1	-5.0
108-39-4	m-cresol	-9.6	-6.0
108-43-0	3-chlorophenol	-9.1	-6.3
108-68-9	3,5-dimethylphenol	-10.9	-7.0
108-86-1	bromobenzene	-8.7	-5.0
108-88-3	toluene	-8.1	-4.8
108-90-7	chlorobenzene	-8.0	-4.8
108-95-2	phenol	-8.4	-5.0
120-12-7	anthracene	-16.3	-10.4
121-14-2	2,4-dinitrotoluene	-13.8	-8.3
122-79-2	phenyl acetate	-10.2	-5.5
129-00-0	pyrene	-18.8	-12.7
141-82-2	malonic acid	-5.8	-3.4
371-41-5	4-fluorophenol	-8.8	-5.9
528-29-0	1,2-dinitrobenzene	-11.6	-6.8
591-20-8	3-bromophenol	-9.7	-6.5
591-50-4	iodobenzene	-9.8	-5.8
62-53-3	aniline	-8.1	-4.1
71-43-2	benzene	-7.1	-3.8
78-79-5	isoprene	-5.6	-2.4
89-71-4	methyl-2-methylbenzoate	-11.6	-7.0
91-20-3	naphthalene	-11.1	-6.8
92-52-4	biphenyl	-12.5	-7.6
93-58-3	methylbenzoate	-11.2	-7.8
93-89-0	ethylbenzoate	-11.9	-7.8
98-95-3	nitrobenzene	-10.2	-6.3

75-07-0	acetaldehyde	-3.8	-2.2
99-65-0	1,3-dinitrobenzene	-13.1	-8.5
98-86-2	acetophenone	-10.1	-6.1
99-99-0	4-nitrotoluene	-11.9	-7.8
90-12-0	1-methylnaphthalene	-13.2	-8.2
99-91-2	4-chloroacetophenone	-11.4	-7.4
123-07-9	4-ethylphenol	-9.9	-6.0
587-03-1	(3-methylphenyl) methanol	-10.0	-6.0
85-01-8	phenanthrene	-16.6	-10.8
100-25-4	1,4-dinitrobenzene	-13.1	-8.4

Table S12. Theoretical intercept values (log$C$) in linear relationships between the MD calculated logK_{cal} and ΔG_{MD} for graphene.

Name	CAS no.	logK_{cal}	ΔG_{MD}	log$C$
nitrobenzene	98-95-3	3.6	-6.3	-1.0
naphthalene	91-20-3	3.9	-6.8	-1.0
1,3-nitrotoluene	99-08-1	4.1	-7.1	-1.0
1-naphthylamine	134-32-7	4.0	-6.9	-1.0
2-naphthol	135-19-3	4.8	-8.0	-1.1
1,3-dinitrobenzene	99-65-0	4.6	-7.8	-1.1
phenanthrene	85-01-8	6.2	-10.0	-1.1
2-chlorobiphenyl	2051-60-7	4.5	-7.4	-0.8
3-chlorobiphenyl	2051-61-8	5.3	-8.7	-1.0
2,4'-dichlorobiphenyl	33284-50-3	4.7	-7.7	-0.9
bisphenol A	80-05-7	3.8	-6.4	-0.8
2,2',5,5'-tetrachlorobiphenyl	35693-99-3	4.5	-7.5	-0.9

2,3',5,5'-tetrachlorobiphenyl	41464-42-0	5.7	-9.1	-0.9
2,3,3',4,4',5-hexachlorobiphenyl	38380-08-4	6.4	-10.0	-0.9
3,3',4,4',5,5'-hexachlorobiphenyl	32774-16-6	9.1	-14.1	-1.2

ΔG_{MD}: kcal/mol; *K*: mL/g; *C*: mL/g

Table S13. Analysis of the conformations of the adsorbates onto BP surface.

Aqueous phase	Gaseous phase
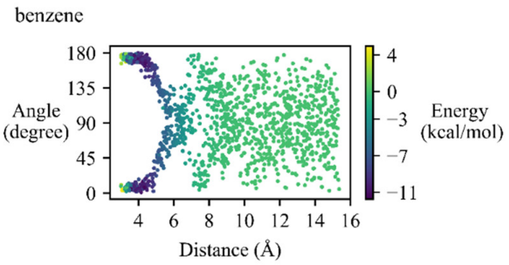	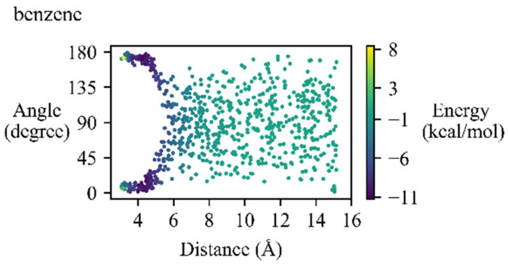
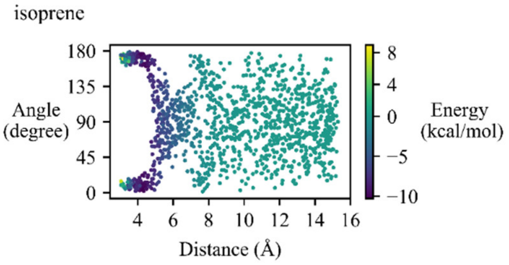	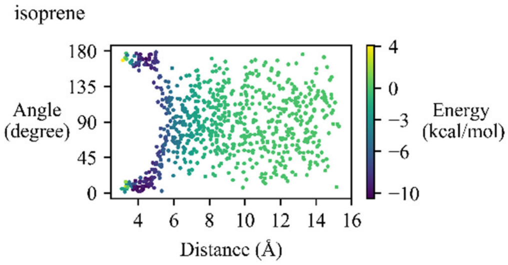
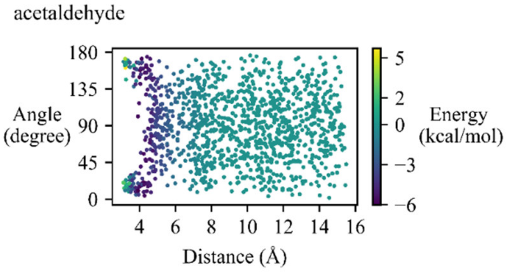	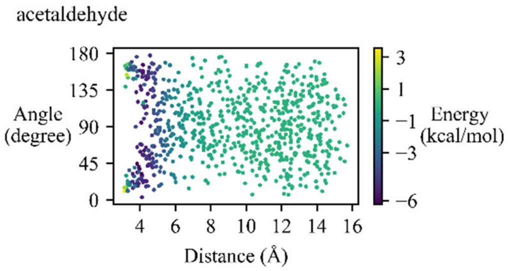

phenanthrene

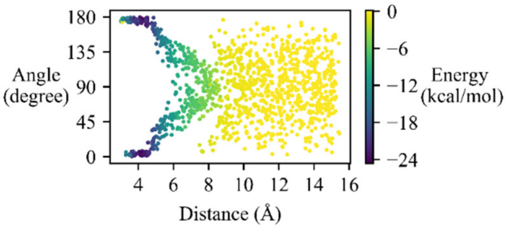

phenanthrene

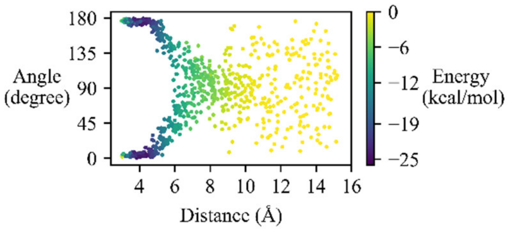

aniline

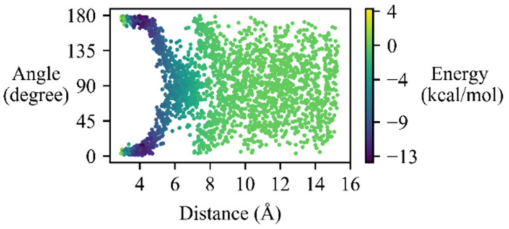

aniline

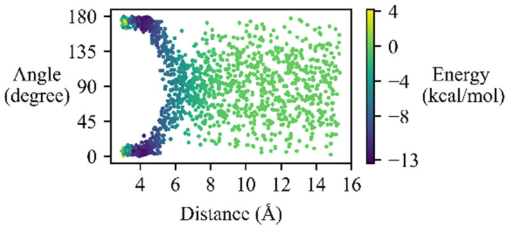

1-methylnaphthalene

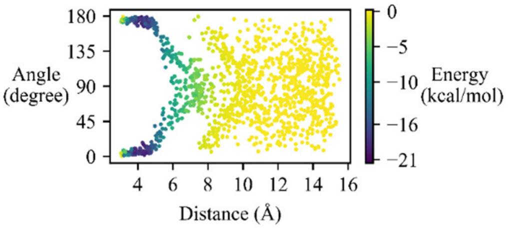

1-methylnaphthalene

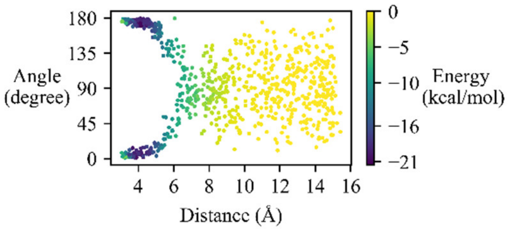

naphthalene

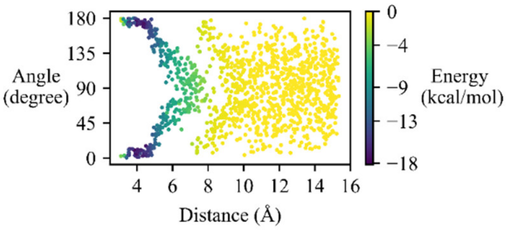

naphthalene

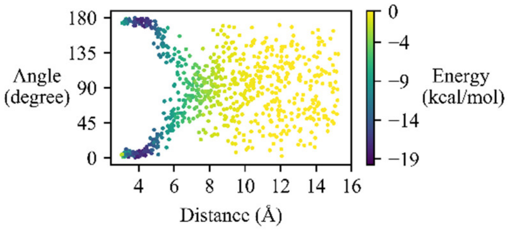

biphenyl

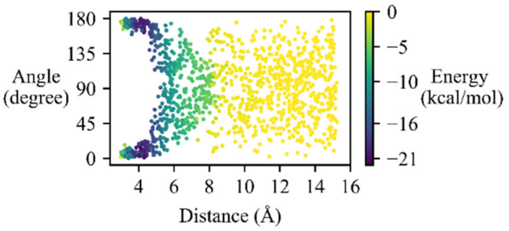

biphenyl

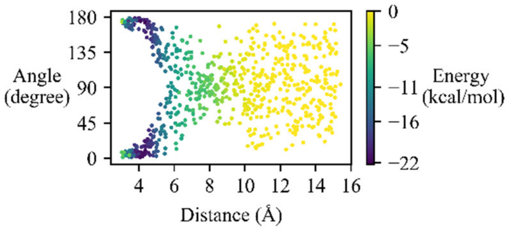

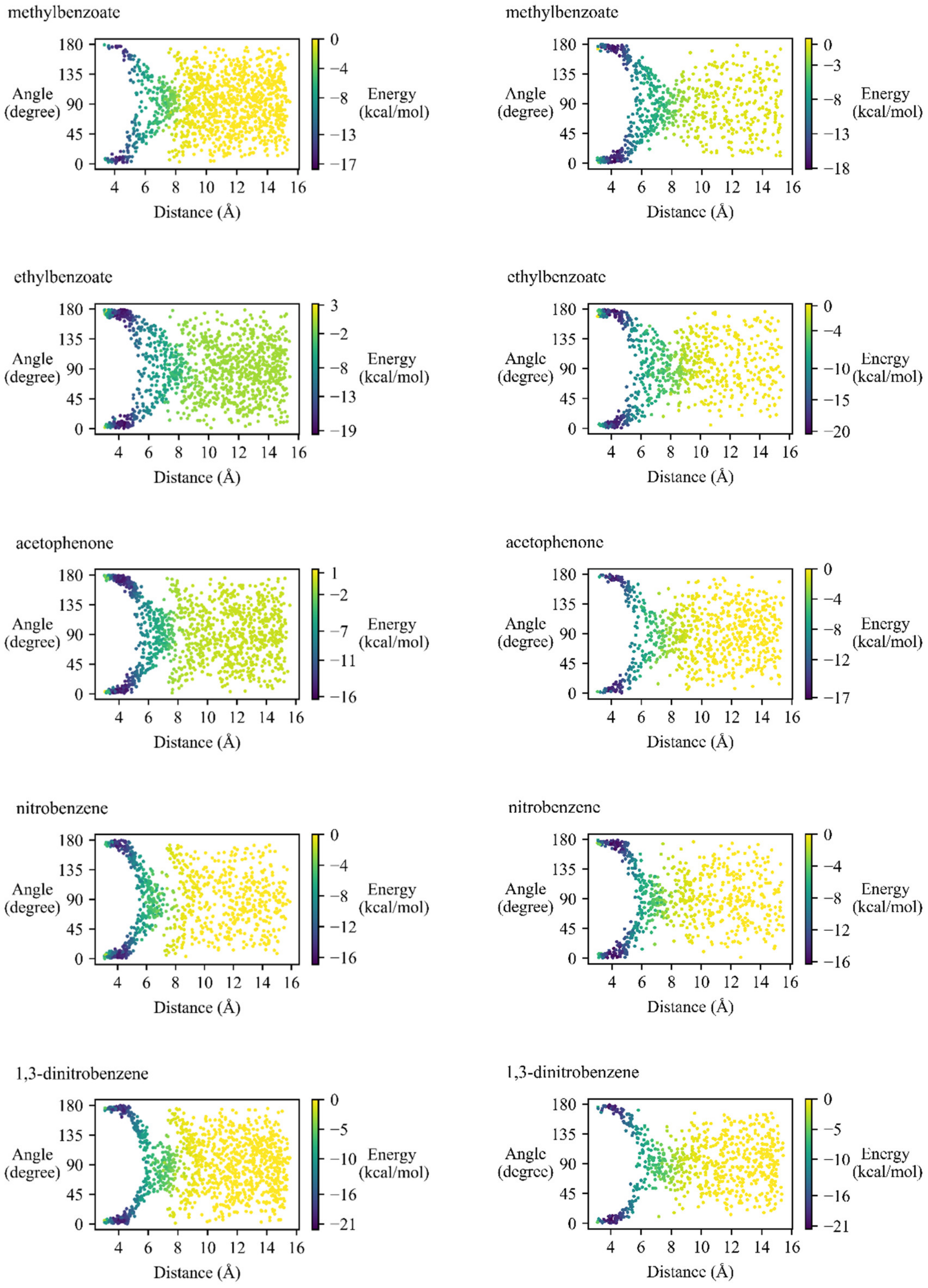
methylbenzoate
Angle (degree)
Distance (Å)
Energy (kcal/mol)
0 −4 −8 −13 −17
methylbenzoate
0 −3 −8 −13 −18
ethylbenzoate
3 −2 −8 −13 −19
ethylbenzoate
0 −4 −10 −15 −20
acetophenone
1 −2 −7 −11 −16
acetophenone
0 −4 −8 −12 −17
nitrobenzene
0 −4 −8 −12 −16
nitrobenzene
0 −4 −8 −12 −16
1,3-dinitrobenzene
0 −5 −10 −16 −21
1,3-dinitrobenzene
0 −5 −10 −16 −21

4-chloroacetophenone

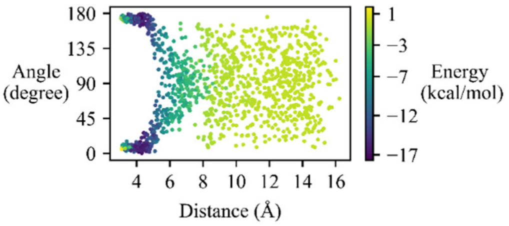

4-chloroacetophenone

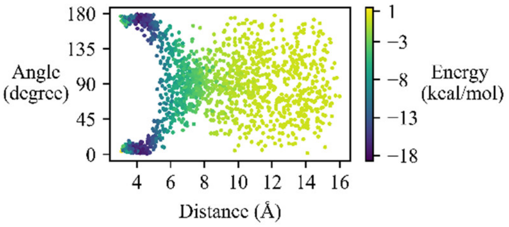

4-nitrotoluene

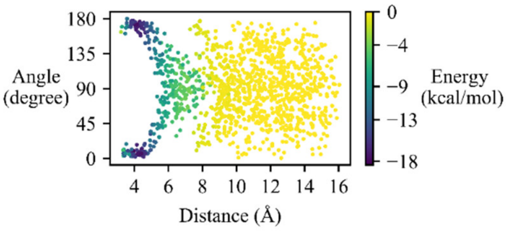

4-nitrotoluene

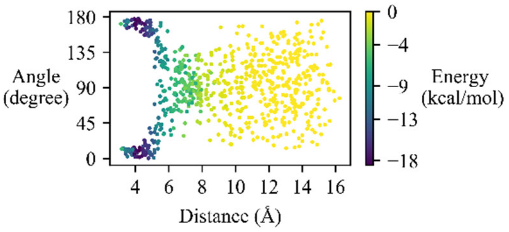

1,4-dinitrobenzene

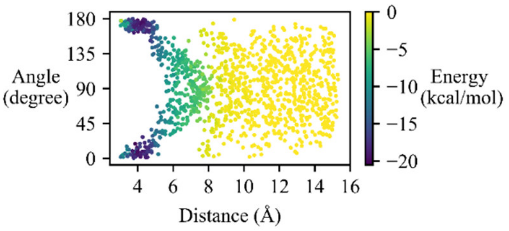

1,4-dinitrobenzene

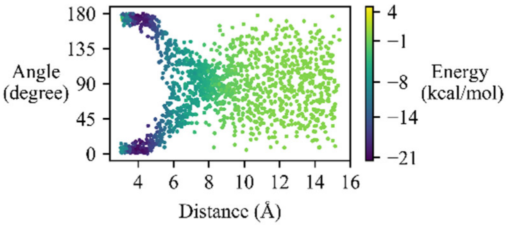

ethylbenzene

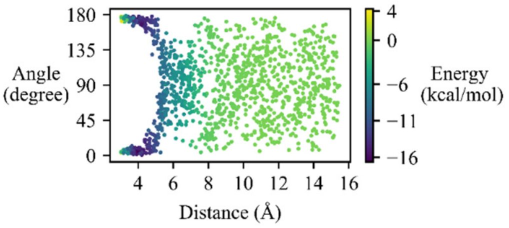

ethylbenzene

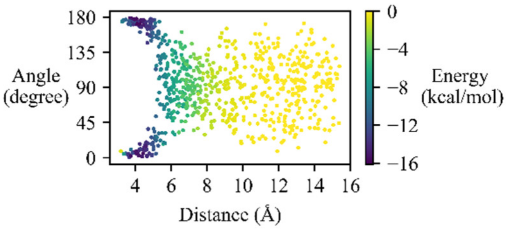

benzonitrile

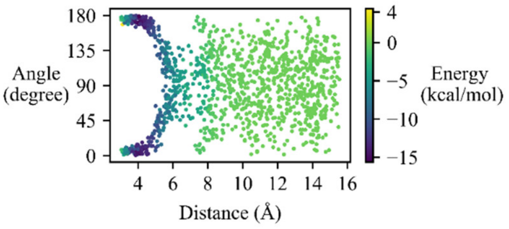

benzonitrile

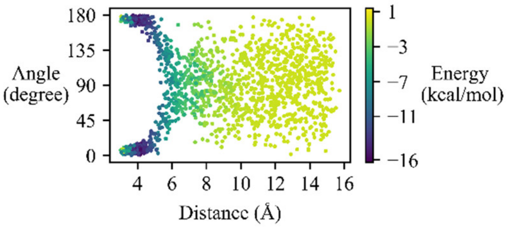

propylbenzene

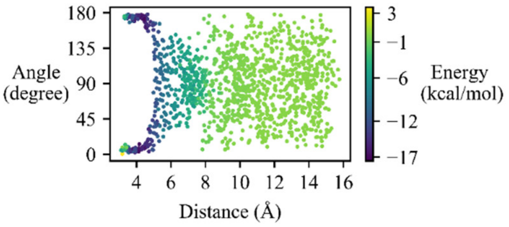

propylbenzene

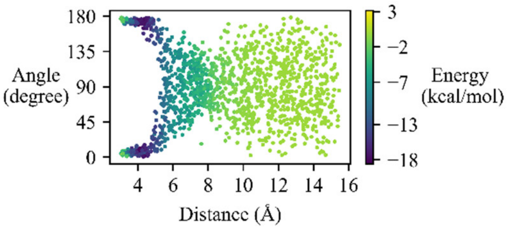

p-xylene

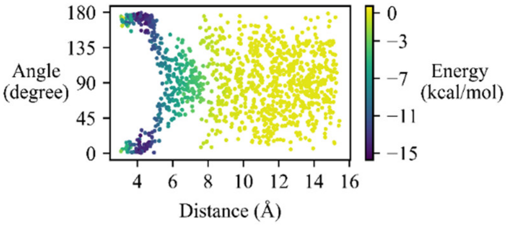

p-xylene

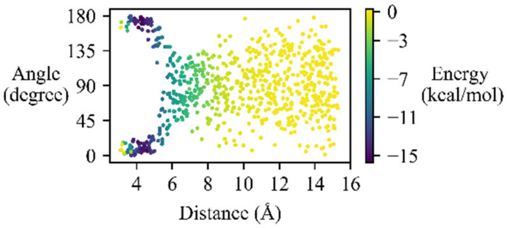

4-chlorotoluene

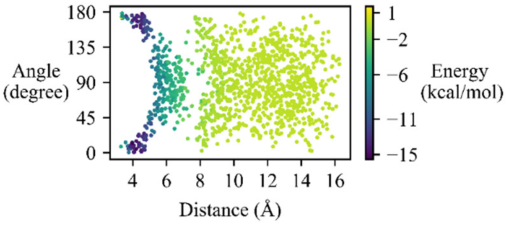

4-chlorotoluene

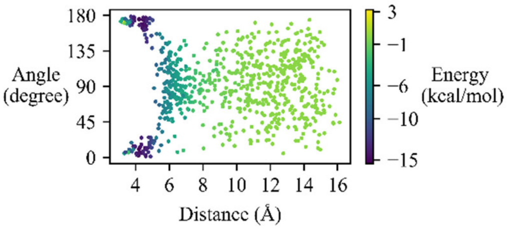

4-chlorophenylamine

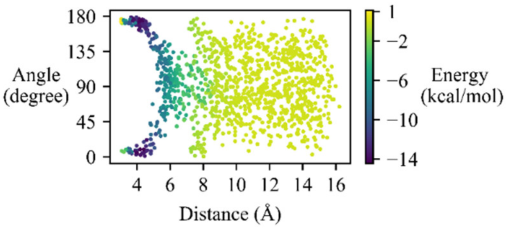

4-chlorophenylamine

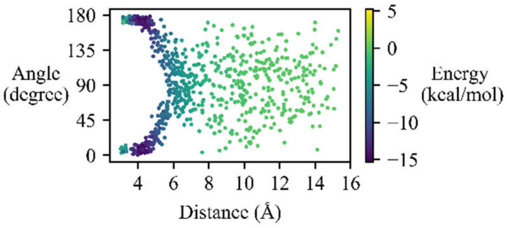

3-chlorophenol

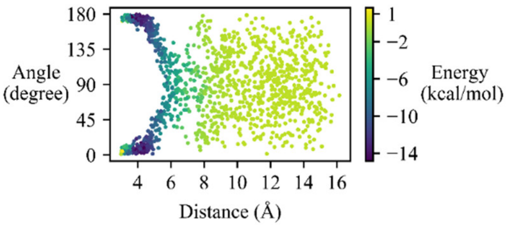

3-chlorophenol

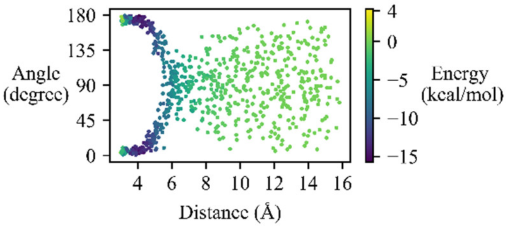

3,5-dimethylphenol

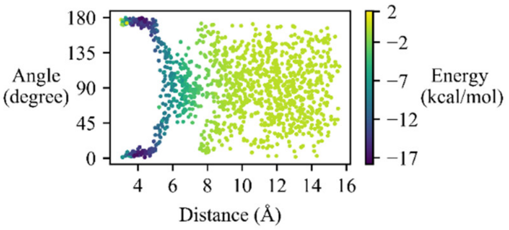

3,5-dimethylphenol

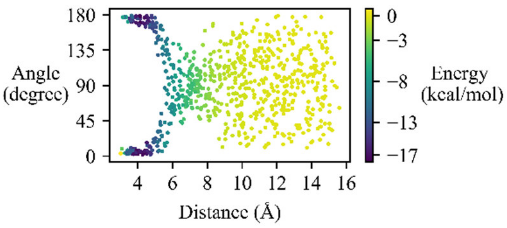

toluene

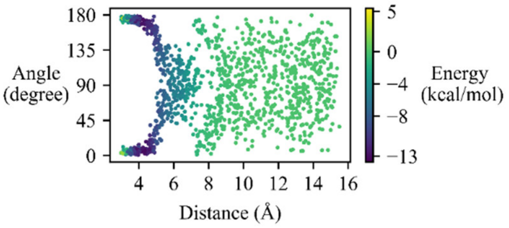

toluene

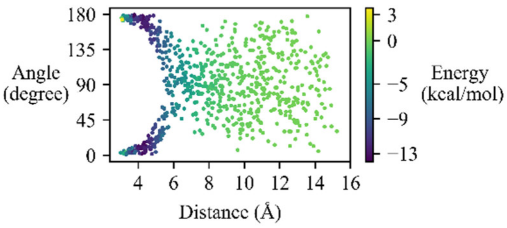

chlorobenzene

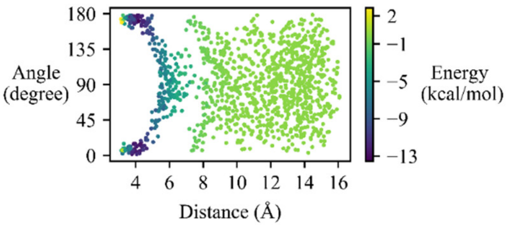

chlorobenzene

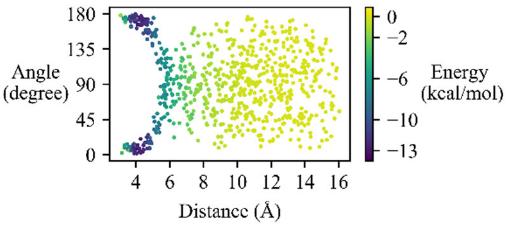

phenol

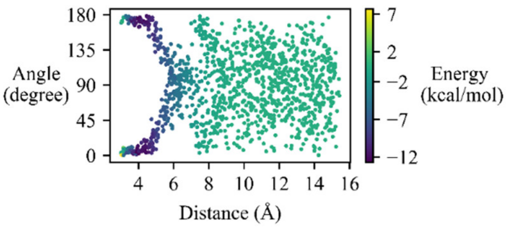

phenol

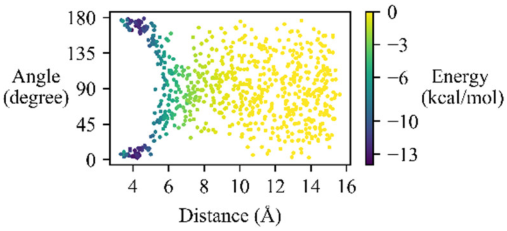

anthracene

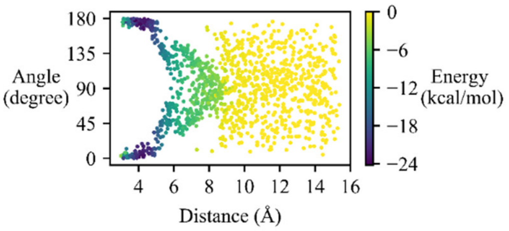

anthracene

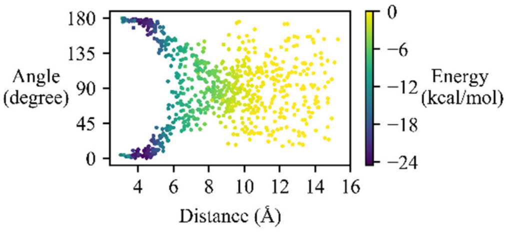

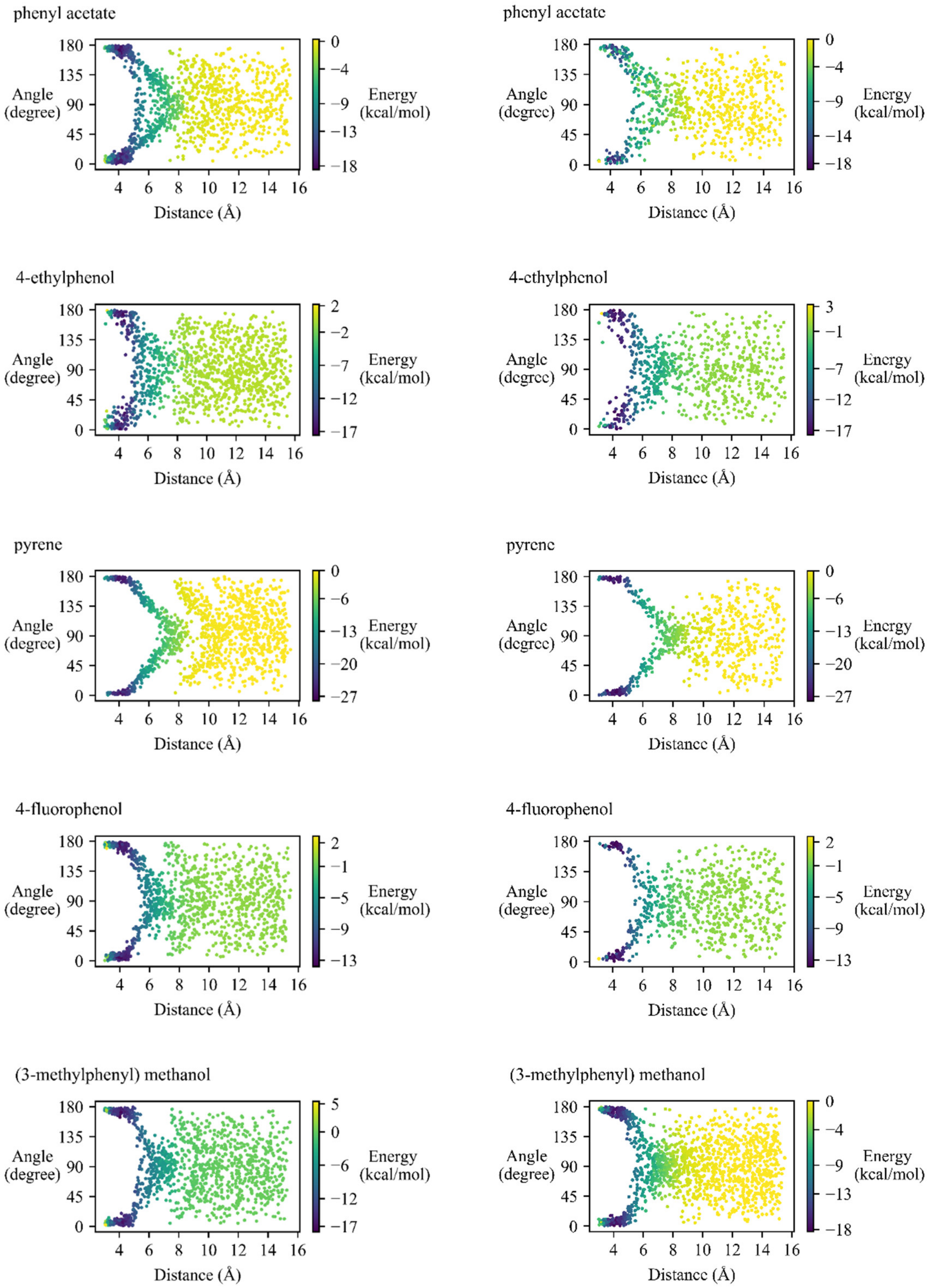
phenyl acetate
Angle (degree)
Distance (Å)
Energy (kcal/mol)
4-ethylphenol
pyrene
4-fluorophenol
(3-methylphenyl) methanol

3-bromophenol

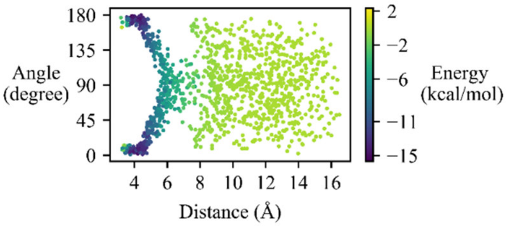

3-bromophenol

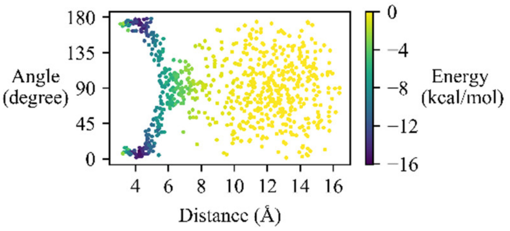

iodobenzene

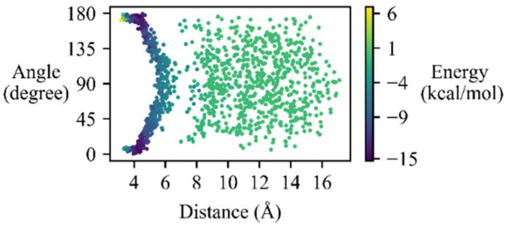

iodobenzene

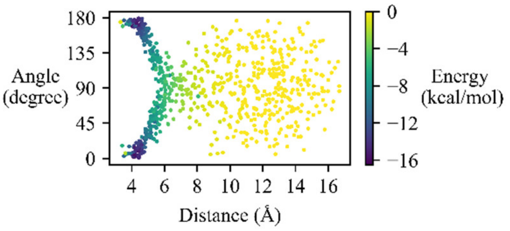

methyl-2-methylbenzoate

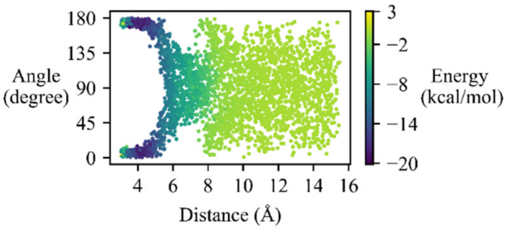

methyl-2-methylbenzoate

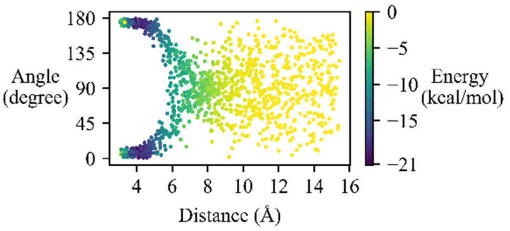

m-cresol

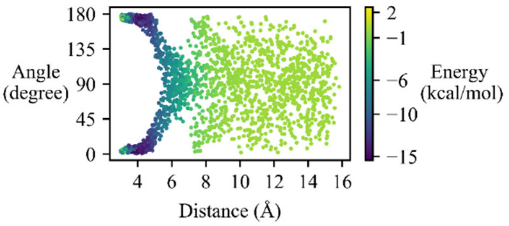

m-cresol

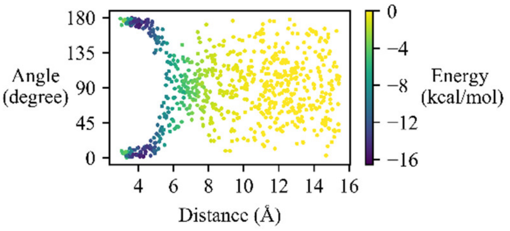

bromobenzene

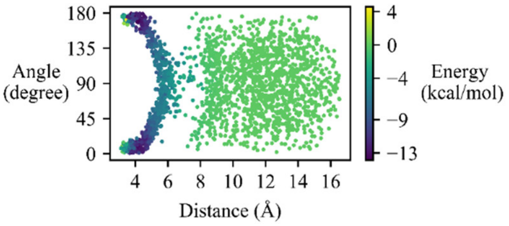

bromobenzene

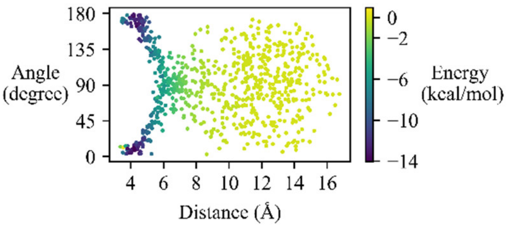

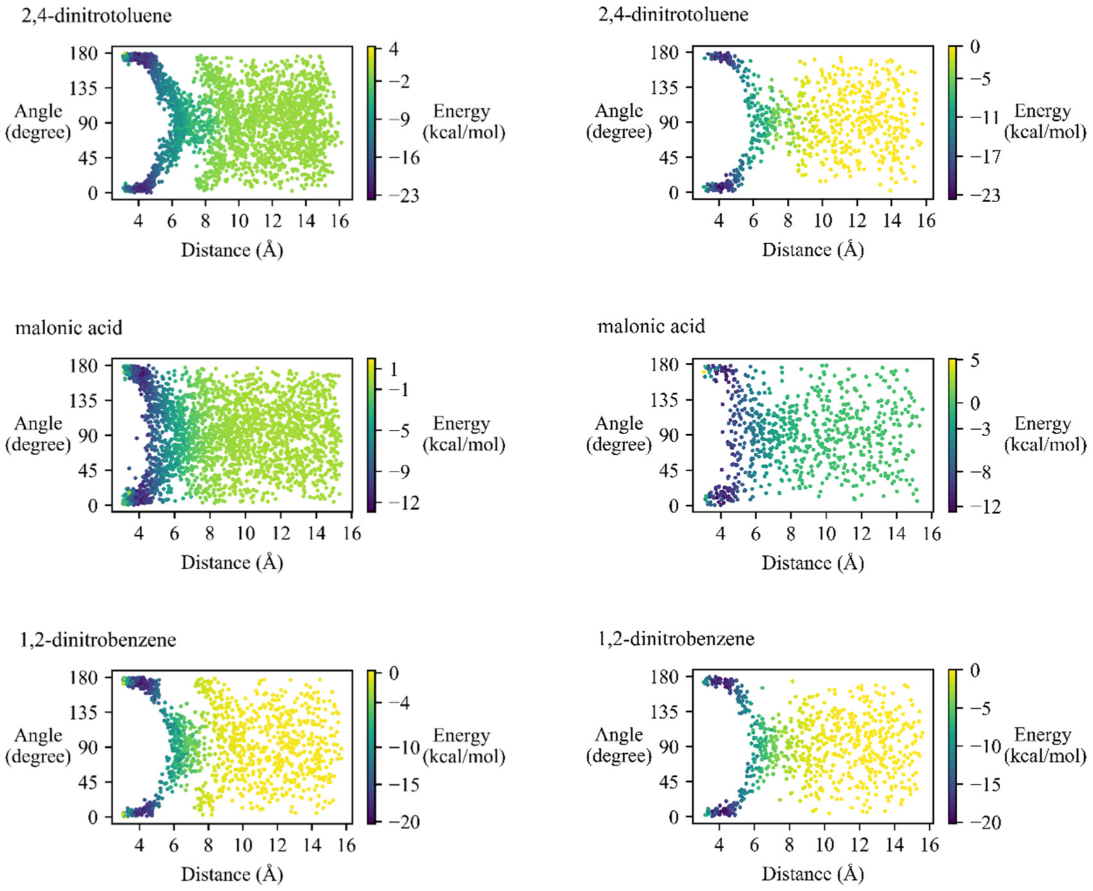

The horizontal ordinates in the figures represent vertical distances between the adsorbates and BP, and the vertical ordinates are dihedral angles between the plane formed by the adsorbates and BP surface. The color bars represent values of interaction energies including electrostatic and van der Waals interactions. The dark color corresponds to strong adsorption.

Table S14. Equilibrium configurations of the adsorbates onto BP surface.

Gaseous phase	Aqueous phase
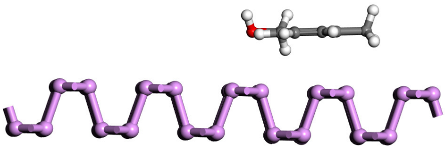	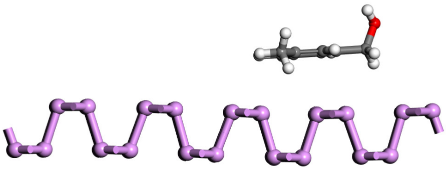
d = 4.599	*d* = 4.603
(3-methylphenyl) methanol	(3-methylphenyl) methanol
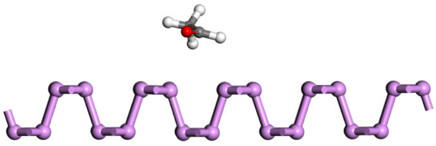	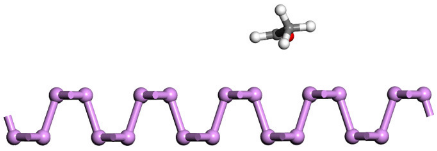
d = 4.436	*d* = 4.401
acetaldehyde	acetaldehyde
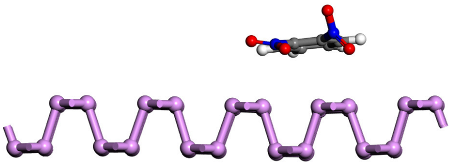	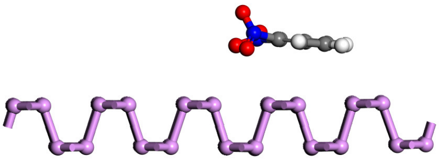
d = 4.584	*d* = 4.553
1,2-dinitrobenzene	1,2-dinitrobenzene
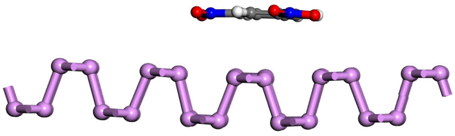	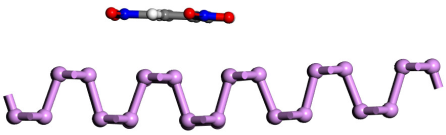
d = 4.439	*d* = 4.434
1,3-dinitrobenzene	1,3-dinitrobenzene

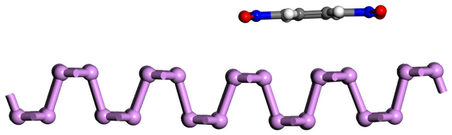

d = 4.481

1,4-dinitrobenzene

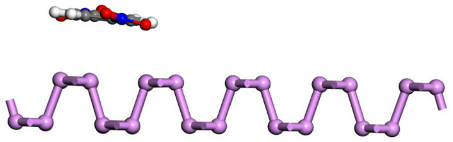

d = 4.466

1,4-dinitrobenzene

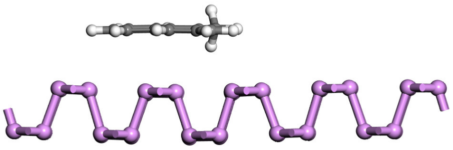

d = 4.481

1-methylnaphthalene

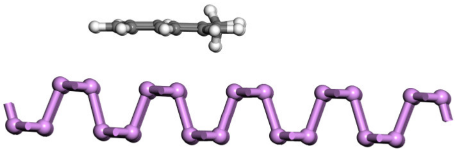

d = 4.511

1-methylnaphthalene

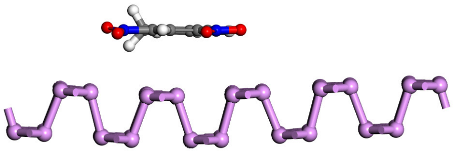

d = 4.497

2,4-dinitrotoluene

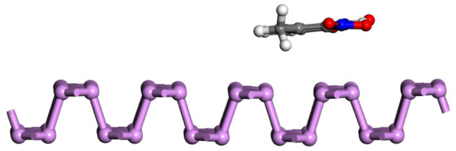

d = 4.515

2,4-dinitrotoluene

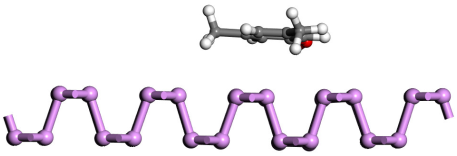

d = 4.498

3,5-dimethylphenol

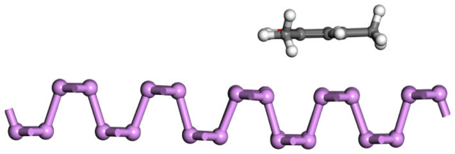

d = 4.456

3,5-dimethylphenol

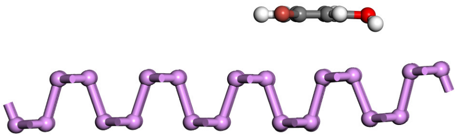

d = 4.440

3-bromophenol

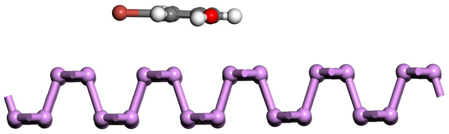

d = 4.424

3-bromophenol

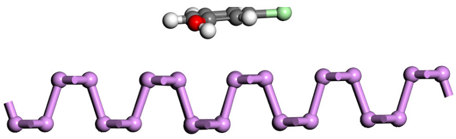

d = 4.393

3-chlorophenol

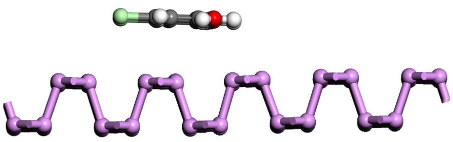

d = 4.465

3-chlorophenol

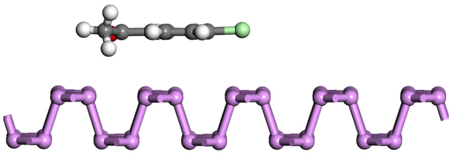

d = 4.524

4-chloroacetophenone

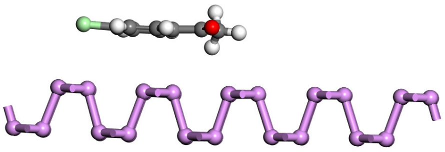

d = 4.526

4-chloroacetophenone

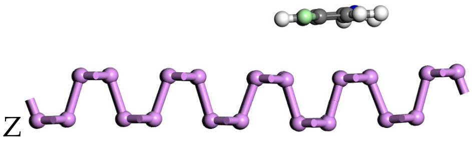

d = 4.447

4-chlorophenylamine

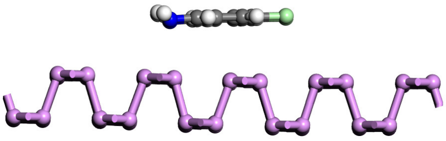

d = 4.513

4-chlorophenylamine

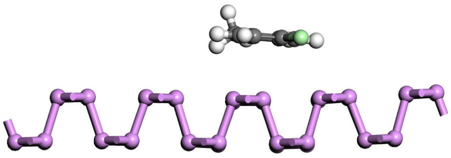

d = 4.551

4-chlorotoluene

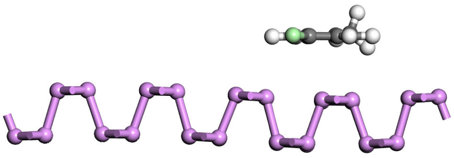

d = 4.613

4-chlorotoluene

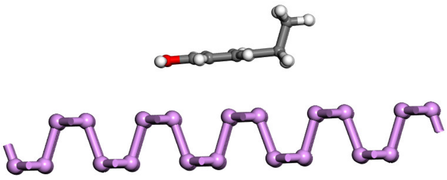

d = 4.773

4-ethylphenol

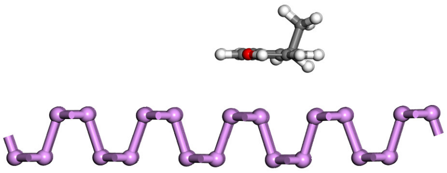

d = 4.762

4-ethylphenol

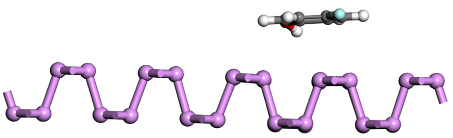

d = 4.427

4-fluorophenol

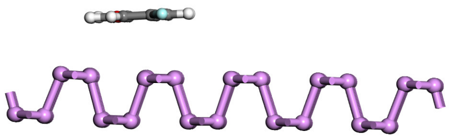

d = 4.479

4-fluorophenol

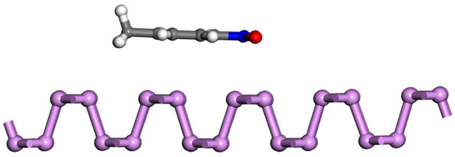

d = 4.490

4-nitrotoluene

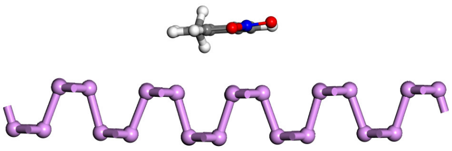

d = 4.476

4-nitrotoluene

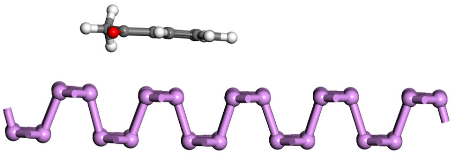

d = 4.485

acetophenone

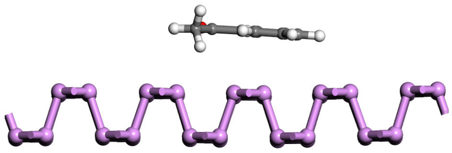

d = 4.493

acetophenone

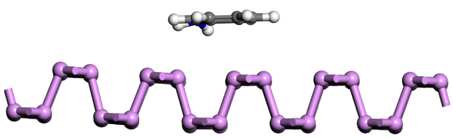

d = 4.393

aniline

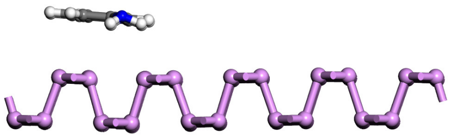

d = 4.396

aniline

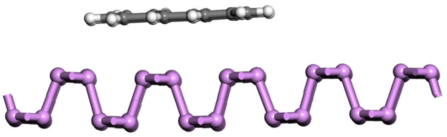

d = 4.453

anthracene

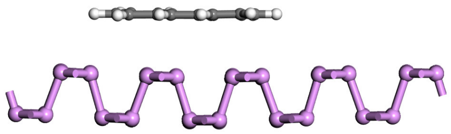

d = 4.466

anthracene

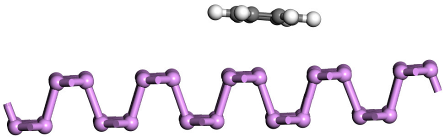

d = 4.440

benzene

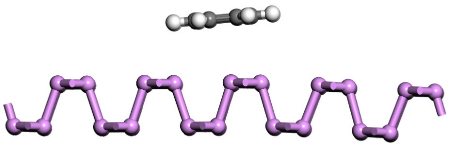

d = 4.438

benzene

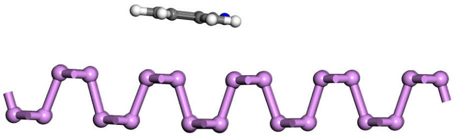

d = 4.470

benzonitrile

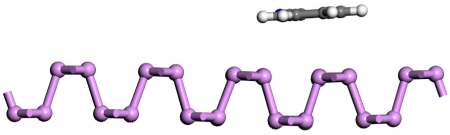

d = 4.493

benzonitrile

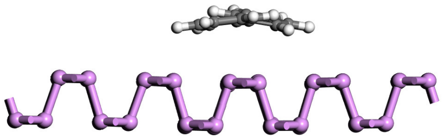

d = 4.596

biphenyl

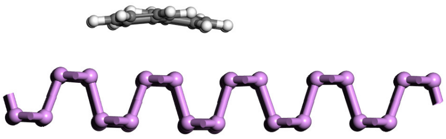

d = 4.547

biphenyl

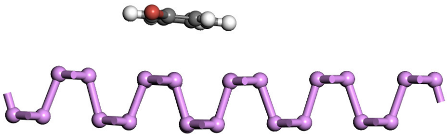

d = 4.523

bromobenzene

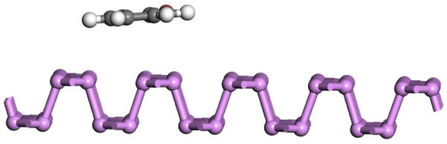

d = 4.437

bromobenzene

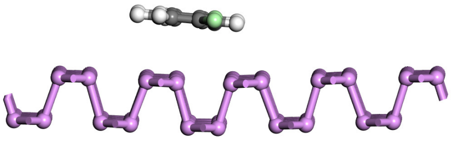

d = 4.490

chlorobenzene

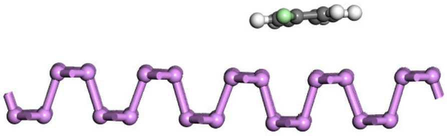

d = 4.449

chlorobenzene

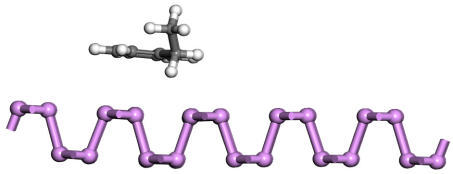

d = 4.729

ethylbenzene

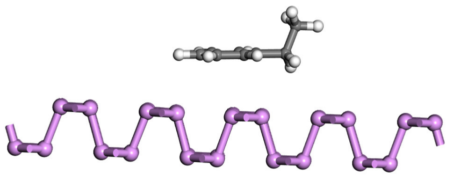

d = 4.692

ethylbenzene

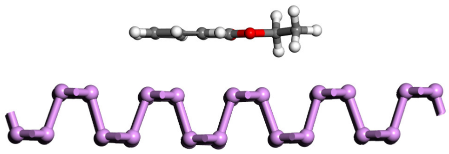

d = 4.516

ethylbenzoate

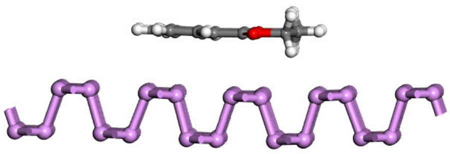

d = 4.554

ethylbenzoate

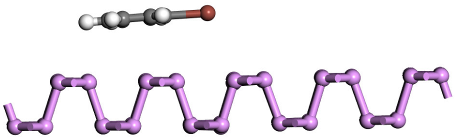

d = 4.484

iodobenzene

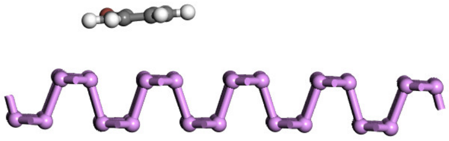

d = 4.501

iodobenzene

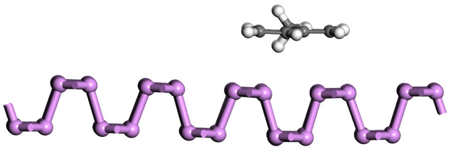

d = 4.509

isoprene

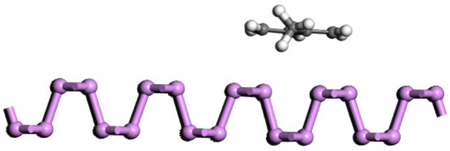

d = 4.422

isoprene

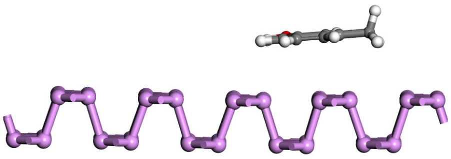

d = 4.432

m-cresol

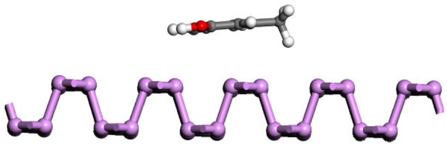

d = 4.478

m-cresol

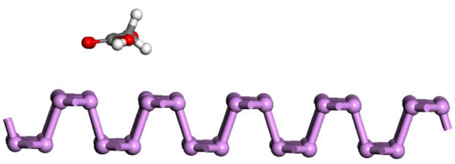

d = 4.426

malonic acid

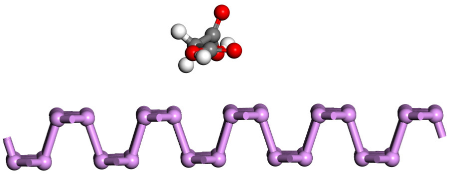

d = 4.528

malonic acid

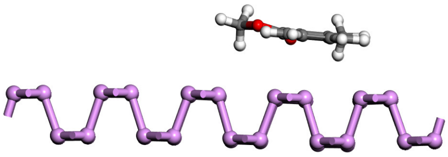

d = 4.505

methyl-2-methylbenzoate

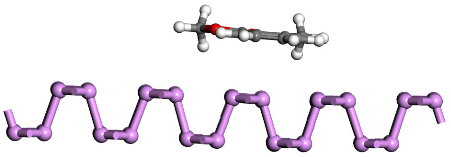

d = 4.503

methyl-2-methylbenzoate

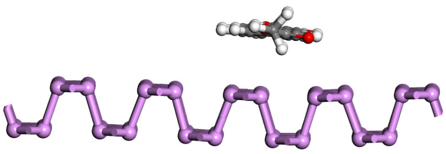

d = 4.467

methylbenzoate

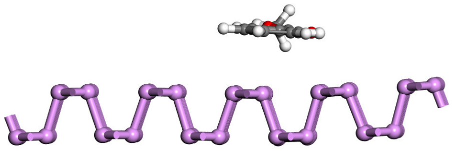

d = 4.489

methylbenzoate

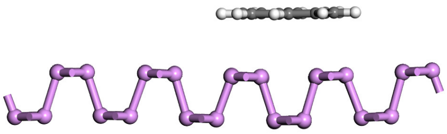

d = 4.537

naphthalene

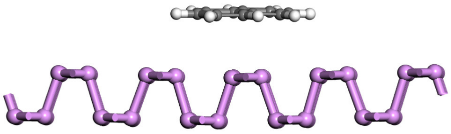

d = 4.350

naphthalene

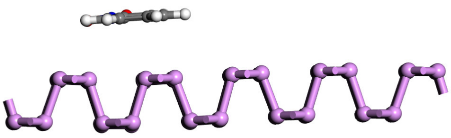

d = 4.394

nitrobenzene

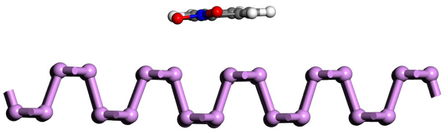

d = 4.434

nitrobenzene

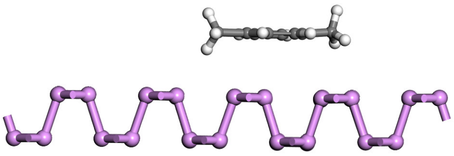

d = 4.619

p-xylene

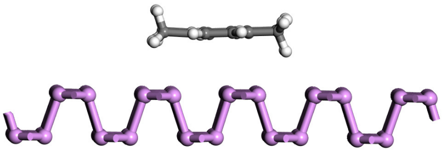

d = 4.589

p-xylene

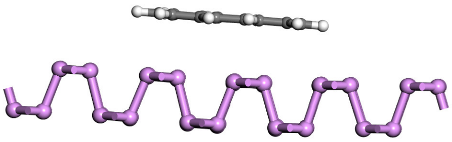

d = 4.516

phenanthrene

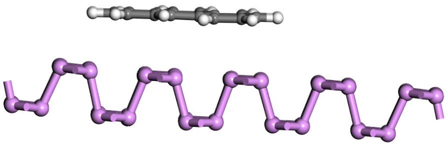

d = 4.473

phenanthrene

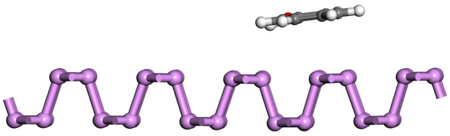

d = 4.434

phenol

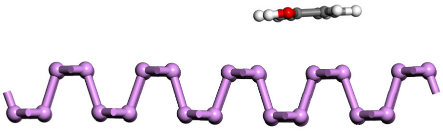

d = 4.381

phenol

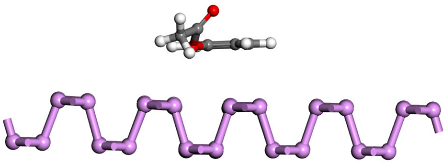

d = 4.610

phenyl acetate

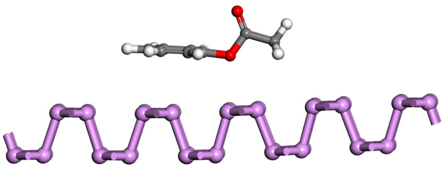

d = 4.562

phenyl acetate

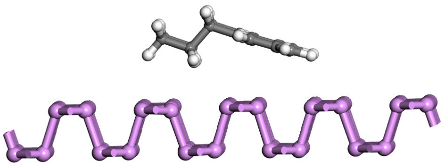

d = 4.638

propylbenzene

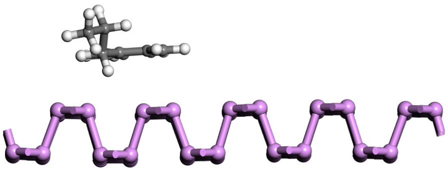

d = 4.899

propylbenzene

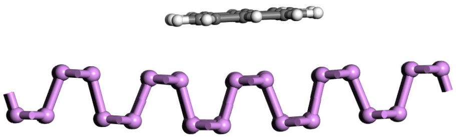<br>*d* = 4.440<br>pyrene	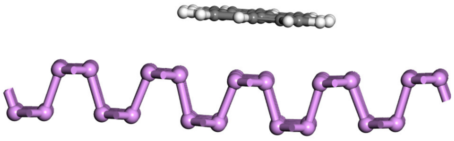<br>*d* = 4.441<br>pyrene
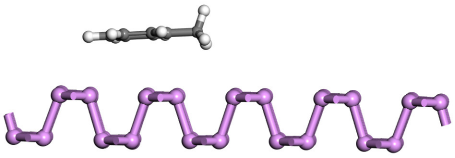<br>*d* = 4.461<br>toluene	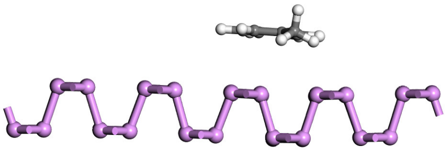<br>*d* = 4.482<br>toluene

d represents the vertical distance between the geometric center of the adsorbates and that of BP.

Table S15. Root mean square deviation (*RMSD*) between the initial and the optimized adsorption configurations of BP.

CAS no.	Name	[a] *RMSD* (nm)	
		Gaseous phase	Aqueous phase
141-82-2	malonic acid	0.02	0.03
78-79-5	isoprene	0.09	0.04
71-43-2	benzene	0.04	0.09
62-53-3	aniline	0.10	0.06
108-88-3	toluene	0.05	0.08
108-95-2	phenol	0.05	0.07
98-95-3	nitrobenzene	0.08	0.08
100-47-0	benzonitrile	0.10	0.09

100-41-4	ethylbenzene	0.08	0.03
98-86-2	acetophenone	0.09	0.06
122-79-2	phenyl acetate	0.06	0.09
93-58-3	methylbenzoate	0.10	0.10
103-65-1	propylbenzene	0.02	0.07
93-89-0	ethylbenzoate	0.10	0.11
528-29-0	1,2-dinitrobenzene	0.07	0.04
99-65-0	1,3-dinitrobenzene	0.15	0.14
100-25-4	1,4-dinitrobenzene	0.13	0.03
99-99-0	4-nitrotoluene	0.05	0.12
106-42-3	p-xylene	0.03	0.03
108-39-4	m-cresol	0.05	0.03
371-41-5	4-fluorophenol	0.06	0.05
123-07-9	4-ethylphenol	0.02	0.05
587-03-1	(3-methylphenyl) methanol	0.03	0.09
89-71-4	methyl-2-methylbenzoate	0.07	0.07
108-68-9	3,5-dimethylphenol	0.05	0.09
591-20-8	3-bromophenol	0.10	0.03
108-43-0	3-chlorophenol	0.05	0.05
99-91-2	4-chloroacetophenone	0.03	0.06
106-43-4	4-chlorotoluene	0.10	0.11
108-86-1	bromobenzene	0.11	0.02
108-90-7	chlorobenzene	0.11	0.08
591-50-4	iodobenzene	0.04	0.07
106-47-8	4-chlorophenylamine	0.10	0.05
121-14-2	2,4-dinitrotoluene	0.18	0.07

91-20-3	naphthalene	0.11	0.09
92-52-4	biphenyl	0.08	0.09
90-12-0	1-methylnaphthalene	0.09	0.06
75-07-0	acetaldehyde	0.02	0.01
85-01-8	phenanthrene	0.10	0.08
120-12-7	anthracene	0.10	0.11
129-00-0	pyrene	0.21	0.14

[a] $RMSD = \sqrt{\frac{\sum_{i=1}^{N}(X_i - Y_i)^2}{N}}$, where N is the number of atoms in BP, X_i is the initial coordinate vector of atom i, and Y_i the coordinate vector of atom i after aligning adsorbates with the initial coordinate vector.

Table S16. Relative contributions of different interactions to the overall adsorption of BP in the gaseous phase.

Term	$\lvert E_{ad} \rvert$	log$K$	[a] log$K$	
			$T\Delta S$	$\lvert E_{ad} \rvert$
eE	7%	0%	-15%	14%
sS	-40%	-17%	61%	-78%
aA	1%	-3%	-5%	2%
bB	11%	6%	-15%	21%
lL	87%	110%	-58%	170%
[b] Sum	66%	96%	-32%	129%

[a] $\Delta G_{MD(gaseous)}$ was decomposed into $T\Delta S$ and E_{ad} using the results of MD and DFT, and $T\Delta S$ and E_{ad} were further decomposed into various intermolecular interactions by pp-LFERs. [b] The sum consists of the five terms with explicit

interactions, and does not include the contributions of the intercept term in the pp-LFERs. The contribution is average fractions of the terms in the sum of the six terms in the pp-LFERs.

Table S17. Adsorption free energy (ΔG_{MD}) and log*K* values for 30 aromatic compounds onto BP and graphene in the aqueous and gaseous phases.

Name	Aqueous				Gaseous			
	BP		Graphene		BP		Graphene	
	ΔG_{MD}	log*K*	ΔG_{MD}	log*K*	ΔG_{MD}	log*K*	ΔG_{MD}	log*K*
pyrene	-12.7	8.1	-12.1	7.7	-18.8	12.5	-18.2	12.4
phenanthrene	-10.8	6.7	-10.0	6.2	-16.6	11.0	-16.4	10.9
anthracene	-10.4	6.5	-10.1	6.2	-16.3	10.8	-16.2	10.9
biphenyl	-7.6	4.5	-7.4	4.5	-12.5	8.1	-12.3	8.2
1-methylnaphthalene	-8.2	4.9	-7.3	4.2	-13.2	8.6	-12.6	8.5
methyl-2-methylbenzoate	-7.0	4.1	-6.4	3.7	-11.6	7.4	-11.0	7.2
ethylbenzoate	-7.8	4.6	-7.7	4.6	-11.9	7.6	-11.6	7.7
naphthalene	-6.8	3.9	-6.8	3.9	-11.1	7.1	-11.2	7.3
3,5-dimethylphenol	-7.0	4.0	-6.8	3.9	-10.9	6.9	-10.1	6.6

(3-methylphenyl)methanol	-6.0	3.4	-6.2	3.5	-10.0	6.4	-9.6	6.2
methylbenzoate	-7.8	4.7	-6.9	4.0	-11.2	7.1	-10.6	6.9
2,4-dinitrotoluene	-8.3	5.0	-7.7	4.6	-13.8	9.0	-13.2	8.8
propylbenzene	-5.8	3.4	-5.3	3.1	-9.9	6.3	-9.4	6.1
1,4-dinitrobenzene	-8.4	5.1	-7.9	4.7	-13.1	8.5	-13.0	8.6
p-xylene	-6.0	3.3	-5.5	3.0	-9.6	6.1	-9.2	5.9
acetophenone	-6.1	3.4	-6.2	3.5	-10.1	6.4	-9.5	6.2
phenyl acetate	-5.5	3.0	-5.6	3.2	-10.2	6.5	-9.7	6.3
1,3-dinitrobenzene	-8.5	5.1	-7.8	4.6	-13.1	8.5	-13.1	8.7
4-ethylphenol	-6.0	3.4	-5.9	3.4	-9.9	6.2	-9.2	6.0
4-nitrotoluene	-7.8	4.6	-7.5	4.4	-11.9	7.6	-11.1	7.3
m-cresol	-6.0	3.3	-5.8	3.2	-9.6	6.0	-9.1	5.9
ethylbenzene	-4.9	2.7	-5.1	2.8	-9.0	5.6	-8.2	5.3
aniline	-4.1	2.1	-3.4	1.6	-8.1	4.9	-7.9	5.0

toluene	-4.8	2.6	-5.1	2.8	-8.1	5.0	-8.0	5.0
nitrobenzene	-6.3	3.6	-6.3	3.6	-10.2	6.5	-9.4	6.1
1,2-dinitrobenzene	-6.8	4.0	-6.2	3.5	-11.6	7.5	-10.7	7.1
benzonitrile	-6.3	3.6	-5.7	3.2	-9.7	6.1	-8.9	5.7
4-fluorophenol	-5.9	3.3	-5.4	3.0	-8.8	5.4	-8.1	5.1
phenol	-5.0	2.7	-4.9	2.6	-8.4	5.1	-7.9	5.0
benzene	-3.8	1.9	-3.5	1.8	-7.1	4.3	-6.5	4.0

ΔG_{MD}: kcal/mol; K: mL/g.

REFERENCES

1. Vanommeslaeghe, K.; Hatcher, E.; Acharya, C.; Kundu, S.; Zhong, S.; Shim, J.; Darian, E.; Guvench, O.; Lopes, P.; Vorobyov, I.; MacKerell, A.D. Charmm general force field: A force field for drug-like molecules compatible with the charmm all-atom additive biological force fields. *J. Comput. Chem.* **2010**, 31(4), 671-690.
2. Azhagiya Singam, E.R.; Zhang, Y.; Magnin, G.; Miranda-Carvajal, I.; Coates, L.; Thakkar, R.; Poblete, H.; Comer, J. Thermodynamics of adsorption on graphenic surfaces from aqueous solution. *J. Chem. Theory Comput.* **2019**, 15(2), 1302-1316.
3. Otyepkova, E.; Lazar, P.; Cepe, K.; Tomanec, O.; Otyepka, M. Organic adsorbates have higher affinities to fluorographene than to graphene. *Appl. Mater. Today* **2016**, 5, 142-149.

4. Lazar, P.; Karlicky, F.; Jurecka, P.; Kocman, M.; Otyepkova, E.; Safarova, K.; Otyepka, M. Adsorption of small organic molecules on graphene. *J. Am. Chem. Soc.* **2013**, 135(16), 6372-6377.
5. Ersan, G.; Apul, O.G.; Karanfil, T. Predictive models for adsorption of organic compounds by graphene nanosheets: Comparison with carbon nanotubes. *Sci. Total Environ.* **2019**, 654, 28-34.
6. Mayne, C.G.; Saam, J.; Schulten, K.; Tajkhorshid, E.; Gumbart, J.C. Rapid parameterization of small molecules using the force field toolkit. *J. Comput. Chem.* **2013**, 34(32), 2757-2770.
